# Development and validation of hierarchical signature for precision individualized therapy based on the landscape associated with necroptosis in clear cell renal cell carcinoma

**DOI:** 10.3389/fphar.2025.1470145

**Published:** 2025-04-04

**Authors:** Gao-Sheng Yao, Jun-Shang Dai, Liang-Min Fu, Juan Lin, Zhi-Ping Tan, Lei Dai, Wei Chen, Jun-Hang Luo, Jin-Huan Wei

**Affiliations:** ^1^ Department of Urology, Sun Yat-Sen University First Affiliated Hospital, Guangzhou, Guangdong, China; ^2^ Department of Obstetrics and Gynaecology, The Chinese University of Hong Kong, Shatin, Hong Kong Special Administrative Region, China; ^3^ Department of Obstetrics and Gynecology, Sun Yat-Sen University First Affiliated Hospital, Guangzhou, Guangdong, China; ^4^ Department of Urology, The Second Xiangya Hospital of Central South University, Changsha, Hunan, China; ^5^ Uro-Oncology Institute of Central South University, Changsha, Hunan, China; ^6^ Department of Pediatrics, The Third Affiliated Hospital, Sun Yat-Sen University, Guangzhou, Guangdong, China; ^7^ Institute of Precision Medicine, Sun Yat-Sen University First Affiliated Hospital, Guangzhou, Guangdong, China

**Keywords:** clear cell renal cell carcinoma, necroptosis, necroptosis scoring system, survival analysis, precise treatment

## Abstract

**Background:**

Increasing evidence is showing that necroptosis has unique clinical significance in the occurrence and development of multiple diseases. Here, we systematically evaluate the role of necroptosis in clear cell renal cell carcinoma (ccRCC) and analyze its regulatory patterns.

**Methods:**

First, we evaluated the expression and enrichment of necroptotic factors in ccRCC using gene set enrichment analysis (GSEA) and survival analysis in the expression profile from The Cancer Genome Atlas (TCGA) to demonstrate the overall mutation of necroptotic pathway genes. Then, we used unsupervised clustering to divide the samples into two subtypes related to necroptosis with significant differences in overall survival (OS) and subsequently detected the differentially expressed genes (DEGs) between them. Based on this, we constructed the necroptosis scoring system (NSS), which also performed outstandingly in hierarchical data. Finally, we analyzed the association between NSS and clinical parameters, immune infiltration, and the efficacy of immunotherapy containing immune checkpoint inhibitors (ICIs), and we suggested potential therapeutic strategies.

**Results:**

We screened 97 necroptosis-related genes and demonstrated that they were dysregulated in ccRCC. Using Cox analysis and least absolute shrinkage and selection operator (LASSO) regression, a prognostic prediction signature of seven genes was built. Receiver operating characteristic (ROC) curves and Kaplan–Meier (KM) analyses both showed that the model was accurate, and univariate/multivariate Cox analysis showed that as an independent prognostic factor, the higher the risk score, the poorer the survival outcome. Furthermore, the predicted scores based on the signature were observably associated with immune cell infiltration and the mutation of specific genes. In addition, the risk score could potentially predict patients’ responsiveness to different chemotherapy regimens. Specifically, Nivolumab is more effective for patients with higher scores.

**Conclusion:**

The necroptosis-related signature we constructed can accurately predict the prognosis of ccRCC patients and further provide clues for targeted, individualized therapy.

## 1 Introduction

Approximately 3% of malignancies in adults are renal cell carcinomas (RCCs) which affect more than 400,000 people worldwide each year (Scelo and Larose, 2018; Bray et al., 2018). Approximately 70% of these patients are diagnosed with clear cell renal cell carcinoma (ccRCC) (Shuch et al., 2015), which is more aggressive and has a higher recurrence rate than other RCC subtypes. Although early stage ccRCC can be treated with surgical removal, nearly one-third of patients eventually have tumor recurrence or metastasis (Jonasch et al., 2014; Choueiri and Motzer, 2017). As a heterogeneous disease, it is unrealistic to accurately predict the prognosis of ccRCC and stratify patients at risk based only on existing clinicopathological features and models. Therefore, finding promising markers in new areas that allow for more accurate prediction is essential to not only improve survival but also optimize individualized treatment regimens for patients with ccRCC.

Multicellular organisms rely on regulatory cell death to maintain metabolic or immune homeostasis. Necroptosis is a caspase-independent form of programmed necrotic cell death, mediated by mixed-lineage kinase domain-like proteins (MLKL) and receptor-interacting protein kinases 1 and 3 (RIPK1 and RIPK3), which is similar to apoptosis in mechanism and necrosis in morphology (Gong et al., 2019). Activation of interferon receptors (IFNRs), toll-like receptors (TLRs), tumor necrosis factor receptors (TNFRs), and pathogen infections are all recognized as necroptosis-inducing signals (Han et al., 2011). In humans, through the recruitment and motivation of PIPK3, the inhibited caspase 8 and RIPK1 initiate the process of necroptosis and further phosphorylate MLKL at Thr-357 and Ser-358 (Sun et al., 2012; He et al., 2009). The phosphorylated MLKL migrates to the cell membrane and destroys its stability and physiological structure, which then leads to diffusion of cellular components within the dead cell (Nicole et al., 2022; Wang et al., 2014; Cai et al., 2014). In 2005, necroptosis was found to promote the occurrence of delayed ischemic brain injury in mice *in vivo* (Degterev et al., 2005). Since then, its multifaceted role in tumorigenesis and anti-tumor response has been continuously investigated (Nicole et al., 2022; Fulda, 2014; Koo et al., 2015). Necroptosis has been proven to be a double-edged sword for cancer, not only preventing cancer progression by regulating cell death (Hockendorf et al., 2016; Geserick et al., 2015) but also promoting tumor development through relevant pivotal factor, such as inducing an immunosuppressive response and stimulating proliferative signals in tumor cells (Strilic et al., 2016; Seifert et al., 2016; Liu et al., 2021). However, the specific impact of the promising field of necrosis on urologic tumors, especially ccRCC, remains unclear.

This necroptotic process involves a large number of molecules and signaling pathways and has unique immunological, physiological, and biochemical consequences. Together, these implicated elements during the pathological process can be effective biomarkers for predicting disease progression. For example, Chen et al. (2022) developed a prognostic model on the basis of three necroptosis-related genes (NRGs) and attempted to accurately relate it to patient outcomes. However, most of the prognostic models proposed for ccRCC are superficial and limited, and therefore the performance achieved is suboptimal. Hence, more comprehensive and effective studies are needed to characterize the regulation of necroptosis in ccRCC.

In this study, based on The Cancer Genome Atlas (TCGA) cohort, we comprehensively characterized the dysregulation of necroptotic genes in ccRCC and explored the main pathways regulated by necroptosis. By screening out the key differentially expressed genes (DEGs), clustered subtypes were delineated, and thus we constructed a necroptosis-related risk signature. In addition, combining clinical parameters and copy number variation (CNV), we comprehensively analyzed the predictive efficacy of prognosis, immune infiltration, and prediction of response to immunotherapy based on this model in patients with ccRCC. The aim of this study is to understand the regulatory mechanisms of necroptosis in cancer cells in more detail so as to provide reliable markers for risk stratification and individualized treatment of ccRCC patients.

**FIGURE 1:**
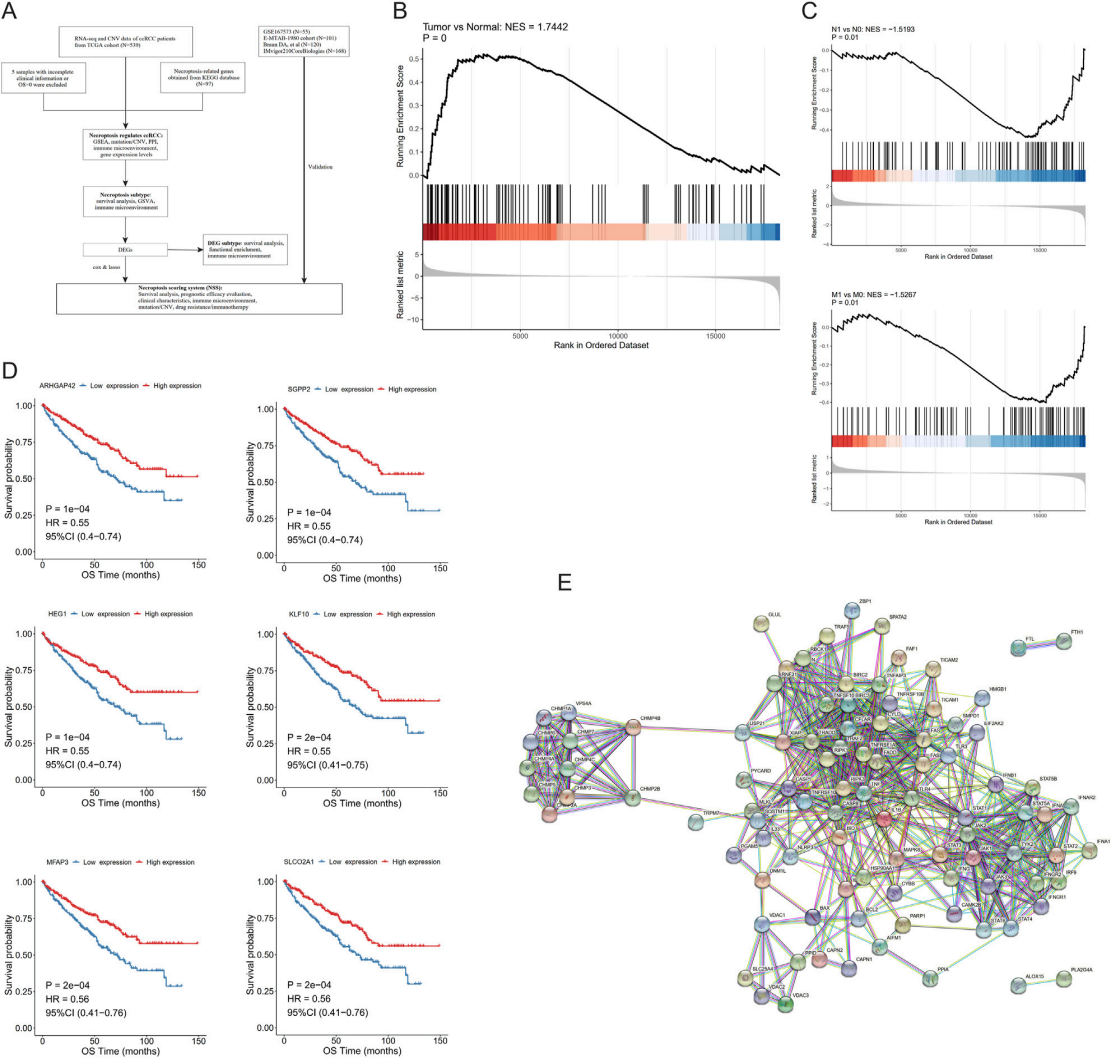
Necroptosis is involved in the development of clear cell renal cell carcinoma (ccRCC). **(A)** Flow chart of this study. **(B)** Gene set enrichment analysis (GSEA) of necroptotic factors between tumor and normal samples. **(C)** GSEA of necroptotic factors in N- and M-stages. **(D)** Kaplan–Meier curves of the most significant six genes in necroptotic factors. **(E)** Interactions between necroptotic factors.

In order to describe the study more intuitively, we displayed the research process in the form of a flow chart in Figure 1A.

## 2 Methods and materials

### 2.1 Data preparation and preprocessing

For the TCGA-ccRCC cohort, both the RNA-seq profiles, which were downloaded in fragments per kilobase million (FPKM) format, and genome CNV data of different samples were obtained from the TGGA database (https://xenabrowser.net/datapages/). We converted data from FPKM to transcripts per million (TPM) format and normalized it to log2 (TPM+1). Data from GSE167573 (Sun G. et al., 2021), including expression matrix and clinical information, were downloaded from the Gene Expression Omnibus (GEO) database (https://www.ncbi.nlm.nih.gov/geo/). Utilized as the validation set, the E-MTAB-1980 dataset was obtained from the ArrayExpress database (https://www.ebi.ac.uk/arrayexpress/).

The surgical specimens of four ccRCC patients who underwent radical nephrectomy without neoadjuvant chemoradiotherapy in the First Affiliated Hospital, Sun Yat-sen University (Guangzhou, China) from 2010 to 2015 were collected for experiments. All patients were accurately informed, and informed consent was signed by all the participants. This study was approved by the Medical Ethics Committee of the First Affiliated Hospital, Sun Yat-sen University, consistent with the principles of the Helsinki Declaration.

Additionally, based on data obtained from Braun et al. (2020) (CheckMate 025, NCT01668784), potential applicability in predicting immunotherapy responses of this signature was evaluated in ccRCC patients. We also validated the necroptosis scoring system (NSS) with the data by using the R package “IMvigor210CoreBiologies” (Mariathasan et al., 2018) to demonstrate the feasibility and generalizability of the signature for application in urologic tumors. Overall survival (OS) was used as the prognostic endpoint, and the Kaplan–Meier (KM) method was used to plot the survival curves to compare survival differences across different groups. In all groups, only patients whose survival data were available and OS > 0 were considered for inclusion in the follow-up analysis. A log-rank test was performed to evaluate the significance of survival differences in a randomized trial, testing the null hypothesis that there is no difference in survival between the groups.

Genes involved in necroptotic pathways were acquired from the Kyoto Encyclopedia of Genes and Genomes (KEGG) database (https://www.kegg.jp/) (Kanehisa and Goto, 2000); 97 genes in total were identified as NRGs (Additional File 1: Supplementary Table S1).

### 2.2 Information of gene expression, mutation, CNV, and chromosome position

Using the R package “limma” (Ritchie et al., 2015), DEGs between ccRCC and control samples were obtained. A Wilcoxon test (Gehan, 1965) was used to analyze the expression differences between groups with different clinical parameters. The R package “maftools” (Mayakonda et al., 2018) was used to depict gene mutations at a holistic level. The R package Circos (Krzywinski et al., 2009) was used to demonstrate the specific distribution of genes on chromosomes.

### 2.3 Enrichment analysis

The R package ClusterProfiler (Yu et al., 2012) was used for gene set enrichment analysis (GSEA) on the basis of the expression profile from the TCGA-ccRCC cohort in order to demonstrate the difference between two groups in necroptotic pathways. The enrichment results of necroptotic pathways were also demonstrated in cancer samples among various clinical parameters, such as age, sex, and stage. Multiple testing correction was performed using the Benjamini–Hochberg method to adjust the false discovery rate (FDR) and reduce false positives that may arise from multiple comparisons.

Using the R package GSVA, gene set variation analysis (GSVA) was performed to investigate whether different subtypes of ccRCC differ in biological processes.

### 2.4 Correlation between necroptotic factors and immune infiltrating cells

In the TCGA-ccRCC dataset, cell-type identification by estimating relative subsets of RNA transcripts (CIBERSORT), single-sample GSEA (ssGSEA), and XCELL were used to calculate immune cell ratios and the correlation between the expression levels of each necroptotic factor. Immune cell abundance was evaluated using Pearson analysis, followed by tumor purity, immune score, and stromal score assessed by estimation of stroma and immune cells in malignant tumor tissues using expression data (ESTIMATE).

### 2.5 Cancer subtypes based on necroptotic factors

The R package ConsensusClusterPlus (Wilkerson and Hayes, 2010) was used for consistent clustering analysis, with Euclidean as the clustering distance and km as the clustering method; 100 repetitions were performed to ensure the stability of classification. The K-elbow method was combined to determine the number of clusters and analyze the survival of samples between different subtypes.

### 2.6 Establishment and verification of the necroptosis scoring system (NSS)

Univariate Cox regression analysis was performed on the DEGs to screen the genes associated with OS, followed by least absolute shrinkage and selection operator (LASSO) regression to eliminate redundancy and construct a prognostic signature which was named “NSS.” The calculation formula follows; *Gene*_*i*_ is the key gene after LASSO regression, and *coef*_*i*_ is its weight.

$$NSS=\sum \left({Gene}_{i}*{Coef}_{i}\right).$$

After calculating the NSS scores of the samples, survival analysis was performed by KM curves, and the predictive power of this prognostic signature was evaluated by receiver operating characteristic (ROC) curves using area under curve (AUC) in both training and testing groups.

CNV data of the high- and low-NSS groups were used to detect the amplification and deletion level using the Genomic Identification of Significant Targets in Cancer 2 (GISTIC2) tool on the GenePattern website (https://www.genepattern.org/#gsc.tab=0). Using this, genes with high frequency mutation of the samples were displayed.

### 2.7 Cell cultures, drug treatment, and transfection assays

Human RCC cell line 786-O acquired from the American Type Culture Collection was cultured in RPMI 1640 (Gibco, United States) containing 1% penicillin–streptomycin (Gibco, United States) and 10% fetal bovine serum (PAN-Seratech, Germany). The cells were cultured under a controlled condition of 37 °C and 5% CO_2_ in a humidified environment.

Necroptotic stimuli, including recombinant human TNF-α, Smac mimetic, and z-VAD (TSZ), can induce TNF-mediated necroptosis (He et al., 2009; Li et al., 2004). They were used for 24 h at 10 ng/mL, 10 nM, and 20 μM, respectively.

Small interfering RNAs (siRNAs) targeting BBOX1, PDK4, SLC16A12, CDH2, TEK, PLS1, SLC40A1, and their negative controls were designed and synthesized by RiboBio (Guangzhou, China) following rigorous quality standards. They were all transfected with Lipofectamine 3000 (Invitrogen, United States). The related sequences are listed in Additional File 2 in Supplementary Table S2.

### 2.8 The extraction of RNA and quantitative real-time PCR (qRT-PCR)

The total RNA was extracted from the cells using TRIzol (Invitrogen, United States) as per the manufacturer’s instructions. Reverse transcription of RNA was performed using the PrimeScript RT reagent Kit (Takara, China), and 2X SYBR Green Pro Taq HS Premix II (AGbio, China) was utilized for qRT-PCR according to the specification. GAPDH was employed as an internal reference for RNA normalization. Primer sequences are presented in Additional File 2 of Supplementary Table S2.

### 2.9 Cell viability assay

We inoculated 2 × 10^3^ cells in 100 μL culture medium on 96-well plates treated with TSZ for 24 h. Then, the CellTiter-Glo^®^ Luminescent Cell Viability Assay kit (Promega, United States) was employed as per the manufacturer’s instructions to assess cell viability. The activity data were normalized to the untreated control cells and presented as a percentage in all samples. The experiments were conducted in triplicate, with three wells repeated each time to ensure repeatability.

### 2.10 Cell proliferation assay

In a cell proliferation assay, 2 × 10^3^ cells were seeded in 100 μL culture medium on 96-well plates, and the relative number of cells was determined at different time points using the Cell Counting Kit-8 (CCK8) assay (Abbkine, China).

### 2.11 Western blot

Proteins were extracted from 786-O cells using RIPA buffer (ThermoFisher, United States) supplemented with proteinase inhibitor (Beyotime, China), and the concentration of each sample was quantified by a Pierce™ BCA protein assay kit (ThermoFisher, United States). These protein samples were then utilized for Western blot analysis. The following antibodies were used in subsequent experiments: anti-RIPK1 antibody (1:1,000 dilution, Abcam, United Kingdom), anti-RIPK3 antibody (1:1,000 dilution, Abcam, United Kingdom), anti-MLKL antibody (1:1,000 dilution, Abcam, United Kingdom), anti-p-MLKL antibody (1:1,000 dilution, Abcam, United Kingdom), anti-GAPDH antibody (1:1,000 dilution, Abcam, United Kingdom), and HRP-conjugated goat anti-rabbit antibody (1:5,000 dilution, Abcam, United Kingdom).

### 2.12 Statistical analysis

All analyses were conducted with R version 4.2.0 (http://www.R-project.org) and relevant packages. Data were analyzed using appropriate standard statistical tests. *P* < 0.05 was defined as statistically significant.

## 3 Results

### 3.1 Necroptosis is involved in the development of ccRCC

After screening the training group, the TCGA cohort contained 524 ccRCC samples and 71 normal control samples in total; the clinical information of its ccRCC patients is shown in Table 1. First, the fold changes (FC) between the two types of samples were calculated, and GSEA analysis was performed by the R package clusterProfiler, which showed that 97 NRGs were enriched more in tumor samples than in normal ones (Figure 1B). Furthermore, applying the same method in tumor samples, we found that these NRGs were significantly enriched in the ccRCC samples with N1 and M1 stage (Figure 1C), while there were no differences among the age, gender, grade, TNM stage, and stage T groups (Additional File 3: Supplementary Figure S1).

**TABLE 1:** Clinical characteristics of 680 eligible patients from TCGA, GSE167573, and E-MTAB-1980 cohorts.

Characteristics	TCGA(N = 524)	GSE167573(N = 55)	E-MTAB-1980 cohort (N = 101)
Age(years)			
≤60	261	NA	44
>60	263	NA	57
*Sex*			
Female	182	33	24
Male	342	22	77
M-Stage			
M0	416	49	94
M1	78	6	7
MX	28	0	0
NA	2	0	0
N-Stage			
N0	239	42	94
N1	16	13	3
NX	269	0	4
T-Stage			
T1	267	37	68
T2	68	9	11
T3	178	7	21
T4	11	2	1
Grade			
G1	13	NA	13
G2	224	NA	59
G3	204	NA	22
G4	75	NA	5
NA	8	NA	0

TCGA, The Cancer Genome Atlas; GSE167573, Gene Expression Omnibus Series 167,573; E-MTAB-1980, cohort, 101 ccRCC, patients from ArrayExpress database.

We then divided these ccRCC samples into high- and low-expression cohorts on the basis of median expression levels of a 97-NRGs expression profile, followed by univariate Cox regression analysis to evaluate the survival disparities between these two groups. Using *P* < 0.05 as the threshold, 50 factors observably correlated with OS were identified (Additional File 4: Supplementary Figure S2) from the cohort screened by univariate Cox regression. KM curves of these 50 necroptotic factors were then drawn, of which six genes with the most significant *P*-value were plotted. It was evident that patients with high expression of these six genes—ARHGAP42, SGPP2, HEG1, KLF10, MFAP3, and SLCO2A1—had better survival (Figure 1D). We attempted to analyze the association between the six-gene mutation and patient survival. In the survival analysis, the mutation group (MUT) was defined as patients having mutations in one or more of these six genes, while the rest were categorized as the wild-type group (WT). Although we intended to perform survival analysis for individual gene mutations, subdividing MUT for each gene would lead to insufficient patient numbers to maintain statistical validity. Hence, we aggregated the mutations of these top-six genes to assess the overall correlation between mutations and survival. Regrettably, our findings did not reveal any statistical significance in this correlation (*P* = 0.078) (Additional File 5: Supplementary Figure S3A).

In addition, the peer-to-peer interactions among these 97 necroptotic factors were analyzed using the Search Tool for the Recurring Instances of Neighboring Genes (STRING) database (https://cn.string-db.org/), and only relationships with high confidence scores (>0.9) were selected. It is evident that most necroptotic factors interact with each other (Figure 1E).

### 3.2 Necroptosis-related gene transcription and alteration in ccRCC

**FIGURE 2:**
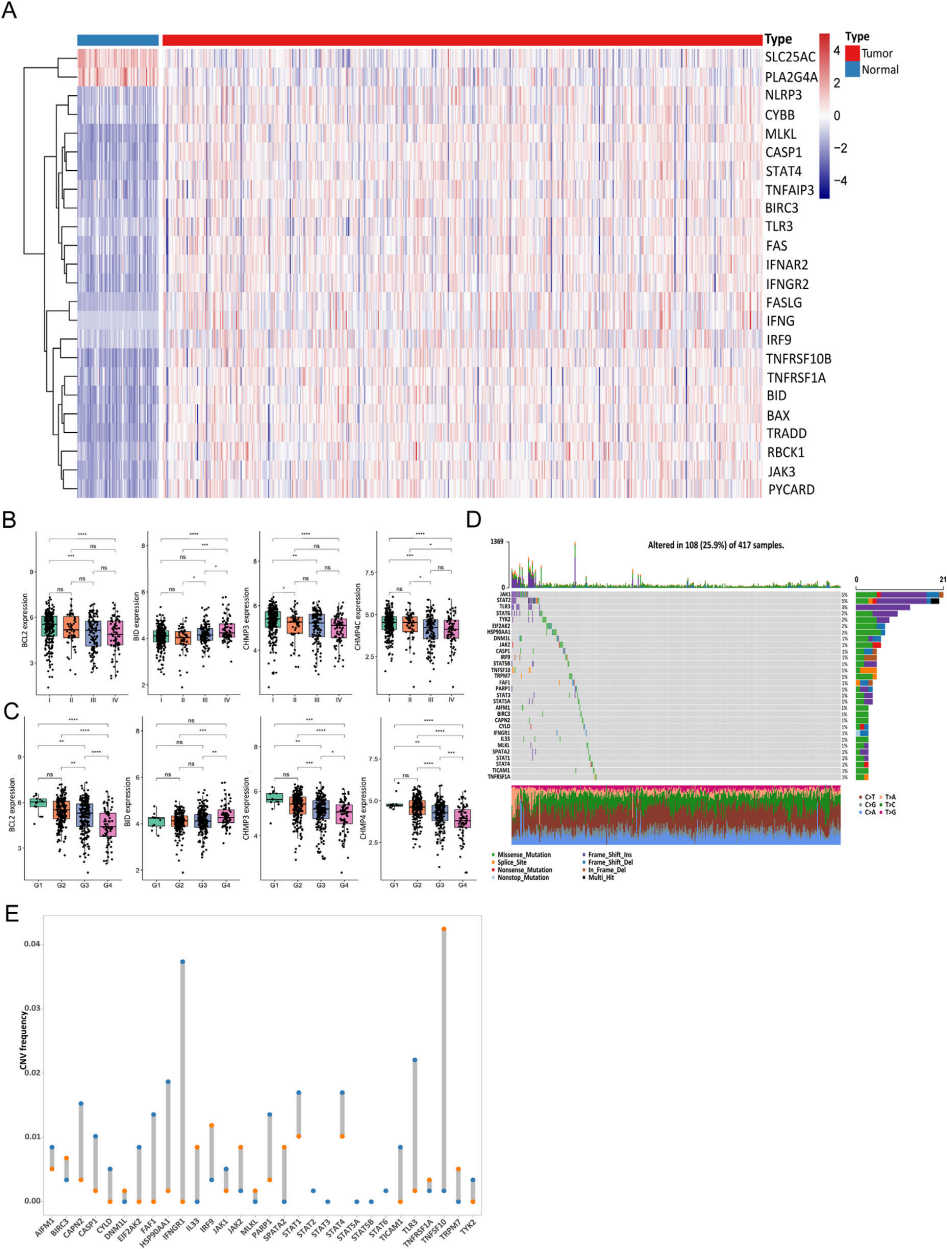
Gene transcription and gene alteration of necroptosis in clear cell renal cell carcinoma (ccRCC). **(A)** Abundance of 24 differentially expressed genes (DEGs) between ccRCC and normal samples. **(B, C)** Differences in expression levels of factors between stage **(B)** and grade **(C)**. **(D)** Mutations of necroptotic genes in The Cancer Genome Atlas (TCGA) cohort. **(E)** Copy number variation (CNV) of the top 30 necroptotic genes. (NS, nonsignificant; *P < 0.05; **P < 0.01; ***P < 0.001; ****P < 0.0001).

According to the expression differences of necroptotic factors in ccRCC and in normal control samples, we screened 24 DEGs with a threshold of |log_2_FC|>1 and *P* < 0.05 (Figure 2A). Similarly, the Wilcoxon test was employed to examine the expression discrepancies of necroptotic factors between groups with different clinical characteristics, and it was found that BCL2, BID, CHMP3, and CHMP4C showed distinct differences among samples at different stages and grades (Figures 2B, C).

Subsequently, mutations of necroptotic factors in the TCGA cohort were summarized. Mutation rates were generally low, and JAK1, STAT2, and TLR3 were the top three genes with mutation rates of 5%, 5%, and 3%, respectively (Figure 2D). In addition, the analysis of the CNV of the top 30 genes showed that most of the factors had copy number amplification or deletion (Figure 2E). To better describe this, we show the location of these 30 necroptotic factors on human genome with a Circos plot (Additional File 5: Figure 3B).

**FIGURE 3:**
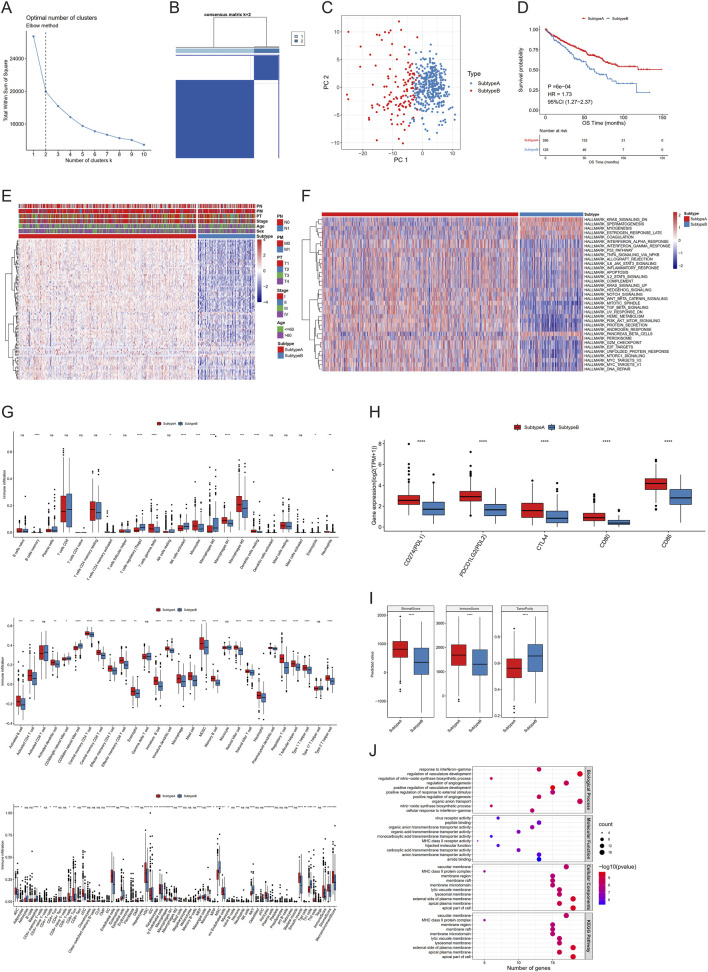
Different regulation models of necroptosis in clear cell renal cell carcinoma (ccRCC). **(A–C)** K-elbow curve **(A)**, sample matrix of consistent clustering analysis **(B)**, and PCA **(C)** of two clusters. **(D–I)** Kaplan–Meier curves **(D)**, necroptotic factor expression **(E)**, gene set variation analysis (GSVA) **(F)**, immune infiltration **(G)**, immune checkpoint expression **(H)**, and estimate scores **(I)** between subtypes. **(J)** Gene set enrichment analysis (GSEA) between differentially expressed genes (DEGs). (NS, nonsignificant; *P < 0.05; **P < 0.01; ***P < 0.001; ****P < 0.0001).

### 3.3 Different regulation models of necroptosis in ccRCC

Firstly, we attempted to type ccRCC samples using necroptotic factors consisting of 24 DEGs—so-called “necroptosis-related subtypes.” Consistent clustering analysis (Euclidean, km, 100 replicates) was performed by the R package ConsensusClusterPlus. The K-elbow method was used to determine the number of clusters, resulting in K = 2, which were then labelled “subtype A” and “subtype B”, respectively. It was clear that the necroptotic factors can effectively classify the samples into two types, and there were remarkable differences in the survival conditions of different subtypes (Figures 3A–E).

To investigate diverse biological behaviors among samples, we conducted GSVA and also compared the differences between the two subtypes, applied with a Wilcoxon test. We obtained 35 pathways exhibiting significant differences between the two subtypes (Figure 3F). To provide a more comprehensive characterization of the variances in immune infiltration between subtypes, CIBERSORT, ssGSEA, and XCELL were utilized to calculate the proportion of immune cells, while a Wilcoxon test was employed to assess inter-group differences. The proportion of diverse immune cells was markedly different between the subtypes (Figure 3G). Subsequently, we used the same method to analyze the expression diversity of different immune checkpoints across subtypes, and all five immune checkpoints showed obvious expression differences (Figure 3H). Finally, ESTIMATE was used to calculate a stromal score, immune score, and tumor purity. Combined with Wilcoxon testing, these three characteristics exhibited significant differences between the two subtypes (Figure 3I).

To further illustrate these two subtypes, we identified 142 DEGs from the comparison between subtypes A and B using a threshold of |log_2_FC|>log_2_ (Shuch et al., 2015) and *P* < 0.05 (Table 2). We found via enrichment analysis that these DEGs were primarily enriched in specific biological processes, including response to interferon-gamma, regulation of vasculature development and regulation of nitric oxide synthase biosynthetic process (Figure 3J). In line with the former, subtypes were further classified on the basis of the 142-DEG expression profile, and DEG-related subtypes were obtained (Additional File 5: Supplementary Figures S3C, D). From the KM curve, better OS was found in patients categorized as DEG-related subtype A (Additional File 5: Supplementary Figure S3E). Then, we ranked the 142 DEGs based on the magnitude of their |log_2_FC| values in descending order and selected the top 30 genes for further differential expression visualization analysis. From the violin plot, these 30 genes were observed to exhibit higher expression levels in DEG-related subtype A than B (Additional File 5: Supplementary Figure S3F).

**TABLE 2:** Differentially expressed genes (142) between subtypes A and B.

Gene					
CYP4A11	SLC27A2	PTPRB	TNFRSF19	MFAP3	AOAH
SLC13A1	BHMT	LRRK2	PODXL	TGFBR2	MNDA
SLC5A8	HRH2	FLT1	S1PR1	CLIC4	HLADRB5
LRP2	QRFPR	VWF	PEG10	ZNF770	HLADPA1
ACE2	SLCO4C1	UBD	SLC16A4	MRC1	SLCO2B1
SCGN	TNFAIP6	PDGFD	APLNR	DDX60	HLADQA1
A1CF	SLC3A1	CPA3	TGFA	CYBB	GBP1
UGT2A3	PRUNE2	SLC1A1	PCDHGC3	SGPP2	MSR1
SLC22A2	AOC1	TEK	LYZ	GPR34	MS4A7
SLC17A3	ZNF366	TLR3	ITGA4	HLADOA	HLADRA
DDC	KL	PLS1	RGL1	CXCL10	F13A1
CUBN	SPON1	SLC4A4	ARHGAP42	THBS1	C3AR1
GSTA2	TFEC	DPP4	AKAP12	SLC40A1	RECQL
CYP2J2	ENPEP	TMEM200A	PPP1R16B	EGFR	LCP1
BBOX1	KDR	SLCO2A1	GIMAP6	KLF10	ITGB1
NPR3	TIMP3	OR2I1P	GBP4	CD84	FPR1
MGAM	APOLD1	TSPAN12	CD93	MPEG1	FCGR3A
SLC16A12	PNMA2	CX3CR1	CP	GJA1	STAT1
GSTA1	GATM	HSPG2	EFNB2	CXCL9	VSIG4
ENPP3	PDK4	CALCRL	TLR7	TLR4	
UGT1A9	C4A	ATP11A	FPR3	CD163	
HAVCR1	CDH2	EGLN3	ETS1	PTPRC	
FMO2	VCAM1	CDH13	FGL2	ITM2A	
CLDN2	PCSK6	PCDH17	ST8SIA4	HEG1	

### 3.4 Construction and validation of NSS

**FIGURE 4:**
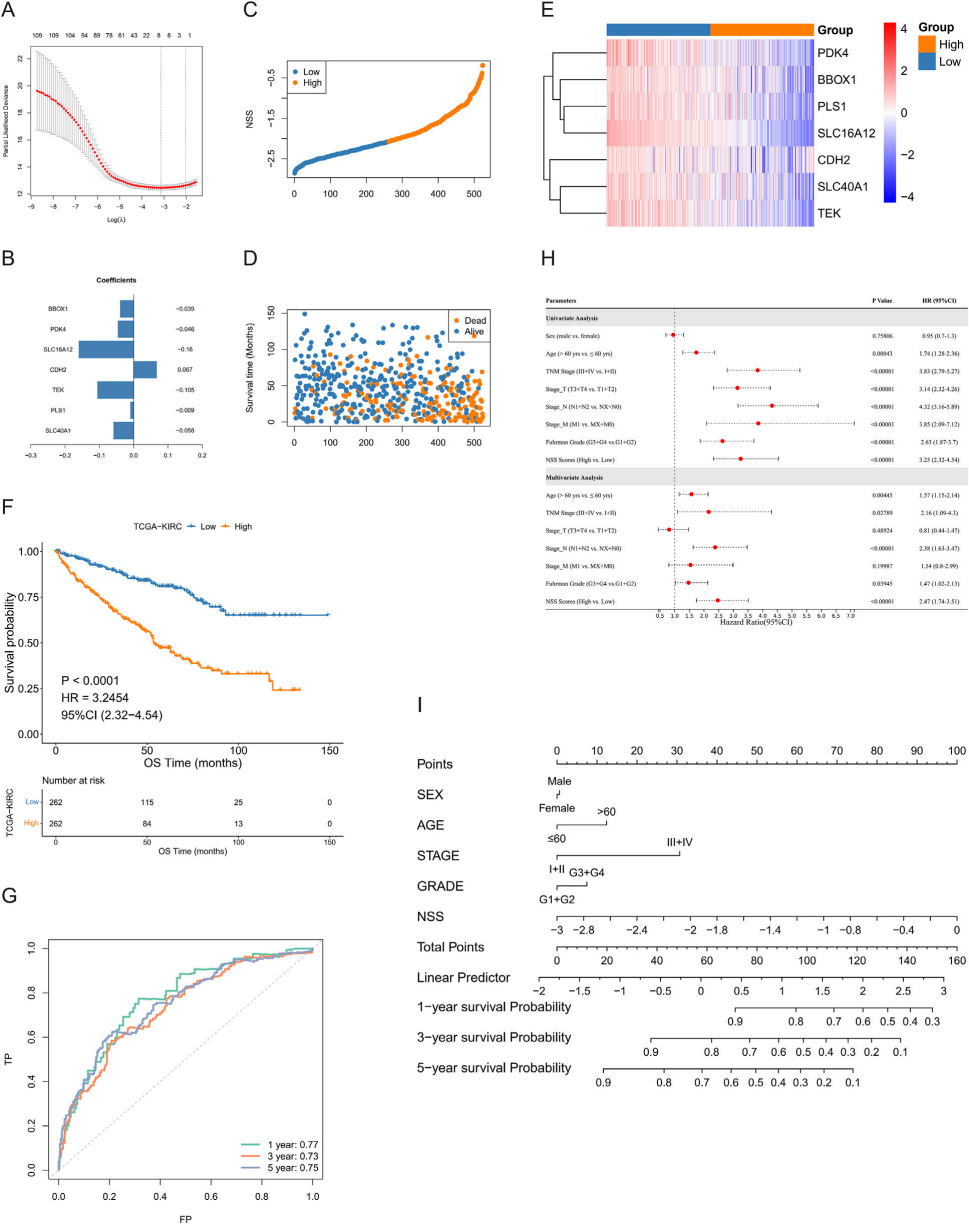
Construction of necroptosis scoring system (NSS). **(A, B)** least absolute shrinkage and selection operator (LASSO) regression **(A)** and weight coefficient of genes **(B)** of model. **(C, D)** Distribution of NSS scores **(C)** and survival time and status of samples **(D)** in the training group. **(E, F)** Expression of model genes **(E)** and Kaplan–Meier curves **(F)** between high- and low-NSS score groups. **(G)** Receiver operating characteristic (ROC) curves of model. **(H)** Univariate and multivariate Cox analyses in training group. **(I)** Nomogram to predict OS for 1/3/5 years.

The DEGs were subjected to univariate Cox regression analysis, and a significance level of *P* < 0.05 was used as the threshold for selecting OS-related genes, resulting in the identification of 111 genes. We then applied LASSO regression to eliminate redundant genes and developed a scoring system (NSS) which consisted of seven genes: PDK4, BBOX1, PLS1, SLC16A12, CDH2, SLC40A1, and TEK (Figures 4A, B). The TCGA-ccRCC cohort was stratified into high and low groups based on the median NSS score of the samples (cut-off = −2.074461), and genes in the signature also presented different distributions among the groups (Figures 4C–E). Through survival analysis, a statistically significant difference in OS was observed between these two groups (Figure 4F). The ROC curve also demonstrated strong predictive performance of the signature, as evidenced by a 1-year AUC exceeding 0.77 (Figure 4G). Similarly, in the testing set GSE167573, the samples are grouped by the NSS median (cut-off = −2.9229). A statistical difference in survival rates between the groups was observed. By analyzing the ROC curve, the signature had better predictive performance, with 1-year AUC valued 0.93 (Additional File 6: Supplementary Figures S4A–E). When we further verified the result in the E-MTAB-1980 cohort (cut-off = −2.773), NSS played a comparably important role, with a more remarkable survival difference and higher AUC value. Notably, the value of AUC demonstrated a consistent increase over time (1-, 3-, 5-year), indicating a progressive enhancement in the signature’s predictive capacity (Additional File 6: Supplementary Figures S4F–J). Therefore, the performance of NSS in both the training and testing sets demonstrated its competence to serve as a survival indicator for ccRCC patients. Moreover, univariate and multivariate Cox analyses were conducted in both the training (Figure 4H; Table 3) and testing (Additional File 6: Supplementary Figures S4K, L; Additional File 7; Supplementary Table S3) sets, showing that the NSS score was an independent prognostic factor associated with adverse survival outcomes. To clarify the link between NSS and clinical parameters, we developed a nomogram that incorporated risk scores and various clinicopathological features to predict overall survival at 1-, 3-, and 5-years (Figure 4I).

**TABLE 3:** Univariate and multivariate Cox regression analyses on OS in the training group.

Parameters	P value	HR	Lower 95%CI	Higher 95%CI
Univariate analysis				
Sex (male vs. female)	0.75806	0.95	0.7	1.3
Age (>60 years vs. ≤60 years)	0.00043	1.74	1.28	2.36
TNM stage (III + IV vs. I + II)	<0.00001	3.83	2.79	5.27
Stage_T (T3+T4 vs. T1+T2)	<0.00001	3.14	2.32	4.26
Stage_N (N1+N2 vs. NX + N0)	<0.00001	4.32	3.16	5.89
Stage_M (M1 vs. MX + M0)	<0.00001	3.85	2.09	7.12
Fuhrman grade (G3+G4 vs.G1+G2)	<0.00001	2.63	1.87	3.7
NSS scores (high vs. low)	<0.00001	3.25	2.32	4.54
Multivariate analysis				
Age (>60 years vs. ≤60 years)	0.00445	1.57	1.15	2.14
TNM stage (III + IV vs. I + II)	0.02789	2.16	1.09	4.3
Stage_T (T3+T4 vs. T1+T2)	0.48924	0.81	0.44	1.47
Stage_N (N1+N2 vs. NX + N0)	<0.00001	2.38	1.63	3.47
Stage_M (M1 vs. MX + M0)	0.19987	1.54	0.8	2.99
Fuhrman grade (G3+G4 vs.G1+G2)	0.03945	1.47	1.02	2.13
NSS scores (High vs. Low)	<0.00001	2.47	1.74	3.51

OS, overall survival; HR, hazard ratio; CI, confidence interval.

**FIGURE 5:**
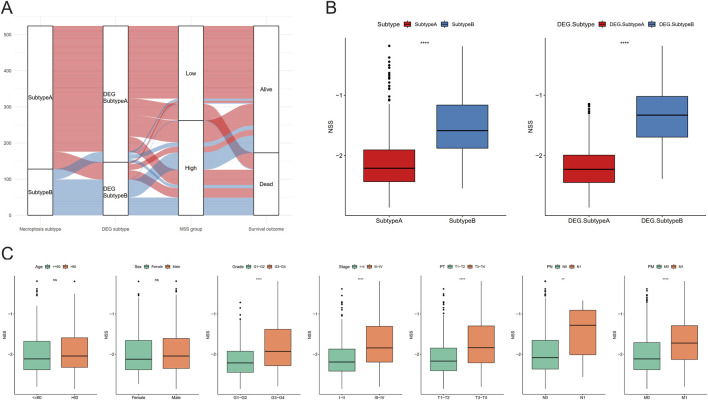
Necroptosis scoring system (NSS) differences between subtypes and clinicopathological features. **(A)** Sankey diagram containing subtypes, NSS group, and survival outcomes. **(B)** NSS differences between the necroptosis subtypes and differentially expressed gene (DEG) subtypes. **(C)** NSS differences between clinical characteristics groups. (NS, nonsignificant; *P < 0.05; **P < 0.01; ***P < 0.001; ****P < 0.0001).

In order to more clearly describe the information of the samples in different categories, we used Sankey diagrams to show the subtypes to which the samples belonged and their corresponding survival conditions (Figure 5A). In addition, differences of NSS between necroptosis- and DEG-related subtypes were also assessed, among which subtype B had higher NSS scores (Figure 5B). We then analyzed the NSS differences among groups with distinct clinical parameters, and statistically different results were found in groups, including grade, stage, stage-T, stage-M, and stage-N, except for age and gender (Figure 5C); this indicated that NSS score can be considered a good indicator for clinical risk stratification.

### 3.5 Analysis of molecular mechanisms involved in NSS

**FIGURE 6:**
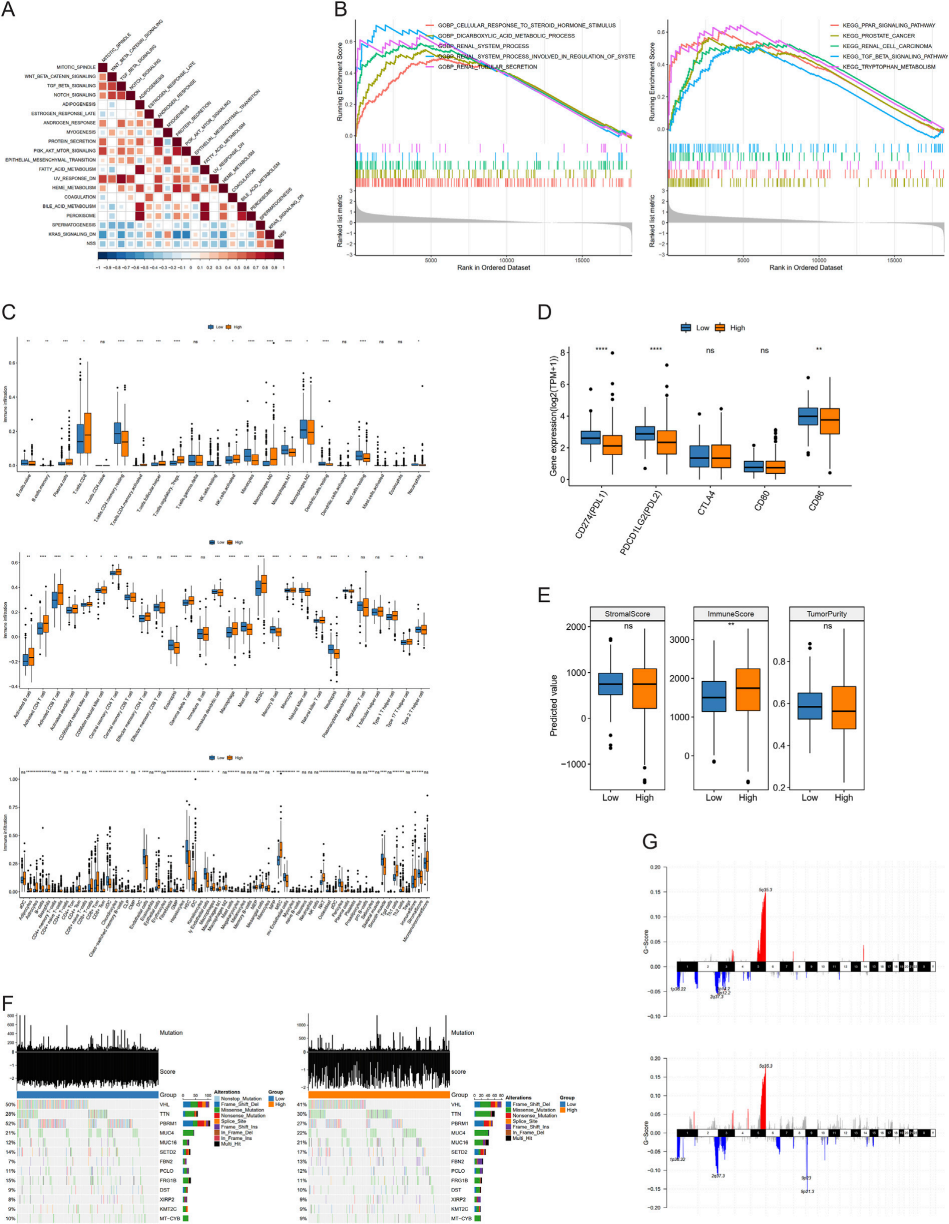
Molecular mechanism analysis involved in necroptosis scoring system (NSS). **(A)** Correlation between NSS scores and enrichment scores of hallmark pathways. **(B)** Gene set enrichment analysis (GSEA) of NSS groups in Gene Otology (GO) and Kyoto Encyclopedia of Genes and Genomes (KEGG). **(C–E)** Differences of immune infiltration **(C)**, immune checkpoint expression **(D)**, and immune score **(E)** between groups. **(F, G)** Mutations **(F)**, amplification and deletion **(G)** in low and high NSS groups. (NS, nonsignificant; *P < 0.05; **P < 0.01; ***P < 0.001; ****P < 0.0001).

To further explore deeper mechanisms of NSS, Pearson correlation analysis between NSS was conducted, and enrichment scores of hallmark pathways were calculated. The results showed remarkable negative correlations between NSS and most hallmark pathways (Figure 6A). Meanwhile, GSEA was conducted in high and low NSS groups, revealing that in the high NSS group, genes with high expression were significantly enriched in biological processes related to renal system function, renal tubular secretion, regulation of the renal system, and other physiological processes (Figure 6B).

Next, we used CIBERSORT, ssGSEA, and XCELL to calculate the percentage of immune cells between groups with different NSS scores, demonstrating that there were indeed differences in immune infiltration between groups (Figure 6C). Then, we employed an identical approach to examine the variations in expression of multiple immune checkpoints between these groups. Three immune checkpoints—PDL1, PDL2, and CD86—displayed distinct expression differences (Figure 6D). Finally, Wilcoxon testing was used to show that the immune score differed between these two groups (Figure 6E).

Beyond that, we identified high-frequency mutated genes between the NSS groups (intersection of top 20; N = 13). The mutation rate of the VHL gene was found to be higher in the low NSS group (50%) than in the high group (41%). Similarly, the mutation rate of PBRM1 was 52% in the low-NSS group whereas it was only 27% in the high group. A Fisher test was used between the groups to screen genes, which had mutation differences with P < 0.05; only PBRM1 and VHL had remarkable differences (Figure 6F). CNV data of these two groups were used to detect amplification and deletion levels. Finally, we found 477 genes with remarkable differences, including CDKN2A, CDKN2B, PTPRD, CSMD3, and DTWD2. (Figure 6G).

### 3.6 Reflection of potential therapeutic strategies from NSS

**FIGURE 7:**
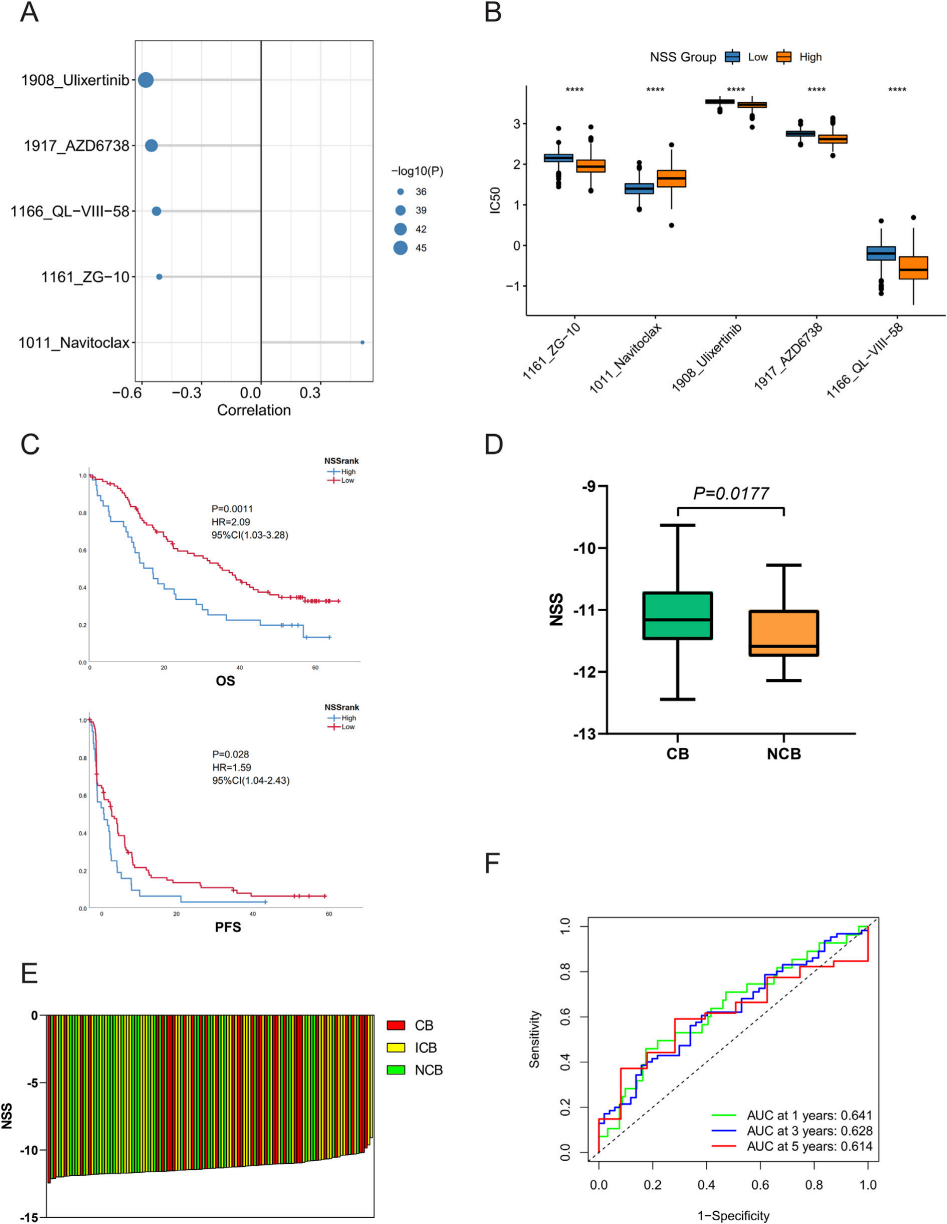
Necroptosis scoring system (NSS) reflects potential therapeutic strategies. **(A)** Five drugs were significantly associated with NSS scores. **(B–E)** Differences of drug resistance **(B)**, overall survival, progression-free survival **(C)**, immunotherapy efficacy **(D, E)** between high- and low-NSS groups. **(F)** Receiver operating characteristic (ROC) curves of NSS in the immunotherapy patient cohort. (*P < 0.05; **P < 0.01; ***P < 0.001; ****P < 0.0001).

To further evaluate NSS for daily clinical guidance, we conducted Pearson correlation analysis between drug resistance and NSS in the Genomics of Drug Sensitivity in Cancer (GDSC) database. Five drugs powerfully associated with NSS were identified, with *P* < 0.05 and |cor|>0.5 serving as a threshold. Drug resistance was quantified using half maximal inhibitory concentration (IC50). Ulixertinib, AZD6738, QL-VIII-58, and ZG-10 showed significant negative correlation with NSS, while Navitoclax showed positive correlation (Figure 7A). The drug resistance analysis of the five objects revealed distinct differences between the high- and low-NSS groups. Navitoclax was clearly higher in the high-NSS group, while the other four drugs showed opposite phenomena (Figure 7B).

Next, we used the patient cohort treated with nivolumab from Braun et al. (2020) to evaluate the efficacy of NSS in predicting immunotherapy potency. In the case of the optimum threshold, OS and progression-free survival (PFS) exhibited significant differences when compared in different groups (Figure 7C), as did the NSS scores of samples in the clinical benefit (CB) versus no clinical benefit (NCB) groups (Figures 7D, E). Furthermore, the ROC curve showed that NSS also played a pivotal role in predicting patient survival (Figure 7F). Finally, when the signature was validated in the IMvigor210CoreBiologies dataset, NSS was also found to have some significance in other cancer types (Additional File 8: Supplementary Figure S5A–E).

### 3.7 Panel of seven genes affecting the viability of ccRCC cells

**FIGURE 8:**
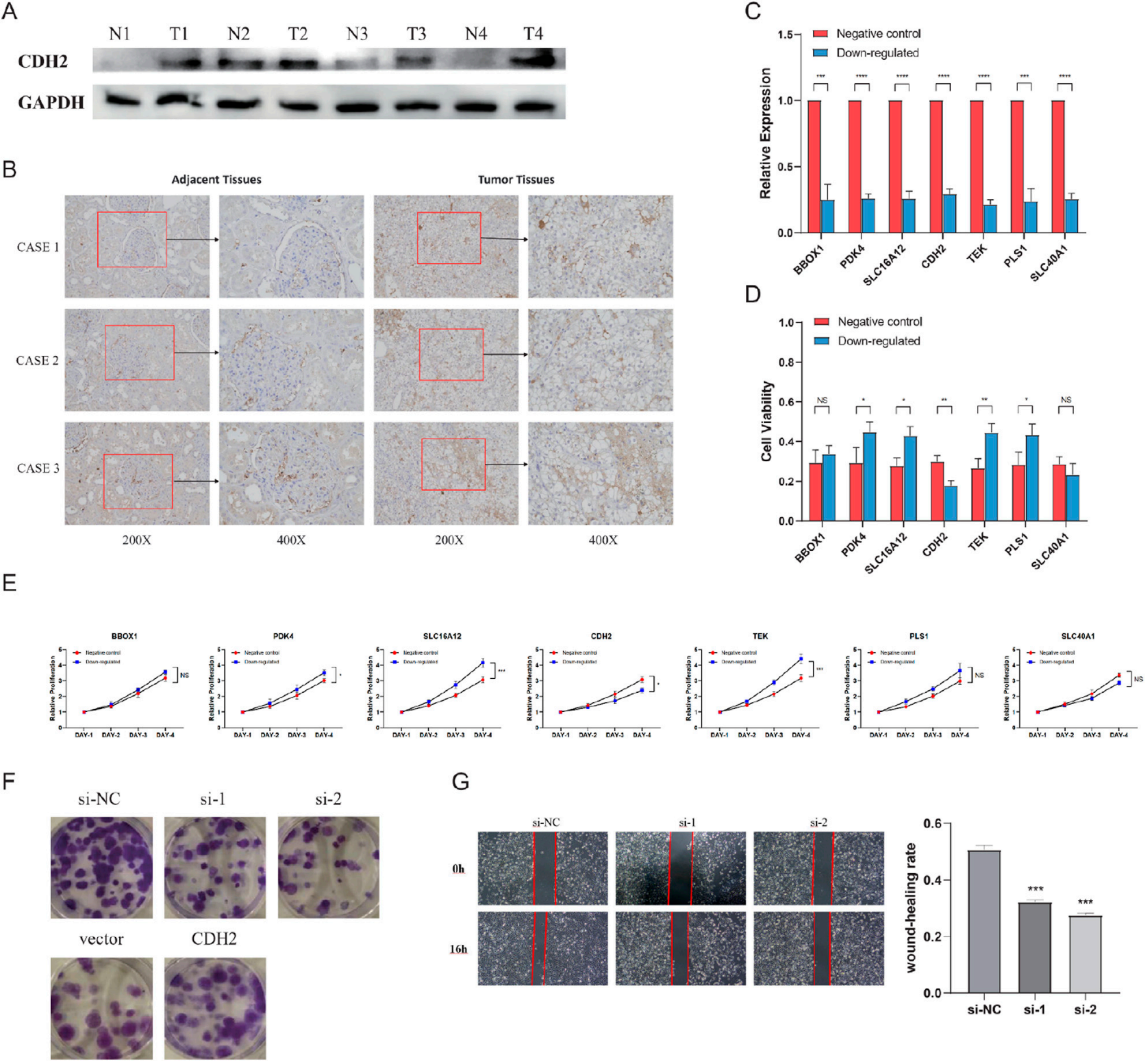
Seven genes affecting the viability of clear cell renal cell carcinoma (ccRCC) cells. **(A)** Immunoblotting analysis of CDH2 protein expression in four pairs of fresh tumors and normal adjacent tissues. **(B)** Comparison of CDH2 protein expression in ccRCC tissues and adjacent tissues via immunohistochemistry (IHC) staining. **(C)** Knockdown efficiencies of seven genes. **(D)** Cell viability after treatment with TNF-α, Smac mimetic, and z-VAD (TSZ). **(E)** Cell proliferation capacity of 786-O transfected with siRNAs or control vectors. **(F)** Proliferative abilities of 786-O cells measured by colony formation after the knockdown and overexpression of CDH2. **(G)** Wound healing assay assessing the migration potential of 786-O cells transfected with CDH2 siRNAs or si-NC. (NS, nonsignificant; *P < 0.05; **P < 0.01; ***P < 0.001; ****P < 0.0001).

We then wanted to validate *in vitro* whether the expression of these seven genes was consistent with the previous bioinformatics analysis. First, CDH2, the only gene with a positive coefficient value in the NSS, was noticed, and it was selected for validation of protein expression between tumor and adjacent non-tumor tissues. The results confirmed that CDH2 was significantly overexpressed in tumor tissues (Figures 8A, B). To investigate the involvement of this seven-gene NSS in ccRCC cell necroptosis, RNA interference (RNAi) to silence their expression in 786-O cells was applied. The knockdown efficiencies were evaluated by qRT-PCR analysis in transformed cells (Figure 8C). We firstly downregulated these seven genes in 786-O cells, then used TSZ to induce necroptosis in these cells, followed by an assessment of cell viability. In 786-O cells with CDH2 knockdown, cell viability was significantly downregulated, while viability of those cells with PDK4, SLC16A12, TEK, and PLS1 knockdown was significantly upregulated, indicating the different role that these genes may play in the process of necroptosis (Figure 8D). However, when we further detected the proteins involved in typical necroptosis pathways using Western blotting, no change was found in protein abundance (Additional File 9: Supplementary Figure S6). The full-length gels can be found in Additional File 10: Supplementary Figure S7. In addition, we observed in CCK8 assays that with the knockdown of PDK4, SLC16A12, or TEK, cell proliferation was inhibited, while knockdown of CDH2 can promote cell proliferation (Figure 8E). Further evaluating the role of CDH2 in 786-O cells, we found that CDH2 can effectively promote cell proliferation and migration (Figures 8F, G).

## 4 Discussion

Although more biomarkers have been proposed, the prognostic outcomes of ccRCC patients are quite inconsistent. Therefore, it is essential to build a more effective signature to facilitate in-patient risk stratification, with more accurate prognosis prediction and better application of individualized treatment. Many studies have been carried out on various phenotypes and mechanisms of ccRCC, but the findings of prognostic factors and therapeutic targets are still unstratified. In this study, a comprehensive study with profiles of transcription, mutation, and immune infiltration was conducted which attempted to construct a more valuable signature of the emerging phenotype.

Necroptosis is a form of immunogenic cell death that has recently attracted increased attention. It is characterized by organelle expansion, lysis of membranes, and the release of intracellular components, known as “damage-associated molecular patterns” (DAMPs), and it can lead to secondary inflammation (Khan et al., 2021). Necroptosis has been demonstrated to be closely implicated in the pathogenesis of numerous diseases, including through the activation or regulation of key molecules such as RIPK3 (Nugues et al., 2014; Afonso et al., 2021) and MLKL (Wang et al., 2014; Tan et al., 2021; Jiang et al., 2025; Yu et al., 2025). In colorectal cancer, Jiang et al. (2025) found that although MLKL promotes cell death during necroptosis, the basal autophagy in colorectal cancer cells is also supported, thereby protecting these cells from death. Yu et al. (2025) also explored the regulatory function of the RIPK1/RIPK3/MLKL pathway on necroptosis in pancreatic cancer progression. They demonstrated that the extracellular exocyotosis of p-MLKL can suppress necroptotic activity *in vitro* and *in vivo*. Accordingly, this has been studied extensively in terms of therapeutics (Koo et al., 2015; Niu et al., 2022; Gao et al., 2024; Khan et al., 2024). Gao et al. (2024) developed a necroptosis-related signature in pan-cancer analysis using bulk RNA sequencing data. They found that this signature can predict patients’ responses to ICIs. In addition, Khan et al. (2024) also investigated necroptosis-based subtypes in categorizing glioblastoma patients to predict their responses to immunotherapy and their prognosis. Necroptosis has been widely explored in tumors, presenting two distinct effects (Yin et al., 2024). From our perspective, it is this double-sided role that makes it urgent to solve the regulatory puzzle of necroptosis in ccRCC.

According to our observation, necroptotic factors were found to be enriched in tumor tissue, and the necroptotic status of the tumor was linked to the OS of ccRCC patients. Furthermore, based on CNV data, most of the key molecules were amplified or deleted in tumor tissue. In fact, as expected, analyses based on multiple omics data tended to produce distinct outcomes in different degrees of necroptosis (Chen et al., 2022; Xin et al., 2022). Next, a risk scoring system related to necroptosis with outstanding performance was constructed based on DEGs and epigenetic alterations. There are also superior results when using the testing group for validation of predictive performance.

In the NRG signature exploited by Chen et al. (2022), patients who scored lower tended to have longer OS than those with higher scores. However, the ROC curves of the signature suggested that only considering the NRGs may not be good enough to predict patient prognosis, with 1-, 3-, and 5-year AUC values of 0.707, 0.635 and 0.667, respectively. As an autonomous prognostic factor for predicting the outcome of ccRCC patients, the 1-, 3-, and 5-year AUC value of NSS signature was 0.77, 0.73 and 0.75, respectively. Surprisingly, when the NSS signature was applied to the testing group, its 1- and 3-year AUC value was 0.927 and 0.829, respectively. In the same horizontal comparison, the NSS model built by us is more efficient than some other models, such as that of Zhang et al. (2023) (with 1-, 3-, and 5-year AUC values of 0.732, 0.680 and 0.709, respectively) and Cai et al. (2023) (with 1-, 3-, and 5-year AUC values of 0.73, 0.67, and 0.70, respectively).

Based on these distinct prognostic subtypes, we further investigated treatment strategies. We investigated the correlation between signature and immune infiltration, and the results showed that it could work as a novel biomarker in immunotherapy. We also performed validation analysis in two immune-treated patient cohorts to support our conclusions. From a medication standpoint, based on the calculated IC50, patients with high NSS scores exhibited sensitive chemotherapy responses to ulixertinib, AZD6738, QL-VIII-58, and ZG-10, while patients with low NSS scores were sensitive to Navitoclax. Based on previous experience and studies, some of these drugs have been proven to be useful for cancer treatment. Ulixertinib is a novel extracellular signal-regulated kinase (ERK) 1 and ERK2 inhibitor, and ERK enhances inflammation by interacting with MLKL and promotes ribosomal S6 kinase (RSK) activation by MAPK signaling (Zhao et al., 2019; Peng et al., 2011; Frodin et al., 2000). AZD6738, an ataxia telangiectasia and Rad3-related protein (ATR) inhibitor, induces high loads of replication stress and forces premature mitotic entry, which then drives mitosis in unreplicated genomes in cells, leading to p53-independent (non-apoptotic) cell death (Lecona and Fernandez-Capetillo, 2018; Blackford and Jackson, 2017). QL-VIII-58, as a Torin2 analog, inhibits PI3K/AKT/mTOR signaling, consequently inducing the accumulation of single-stranded DNA and cell death due to mitotic failure or replication catastrophe (Chopra et al., 2020; Liu et al., 2013). ZG-10 is an inhibitor of c-Jun N-terminal kinase (JNK), which positively regulates autophagy to counteract apoptosis by targeting mitogen-activated protein kinase 8 (MAPK8) (Luo et al., 2021; Wu et al., 2019; He et al., 2012). Navitoclax (ABT-263) is a classic B cell lymphoma 2 (BCL2) inhibitor, while BCL2 has been reported to simultaneously inhibit apoptosis, pyroptosis, and necroptosis (Zhu et al., 2016; Shi and Kehrl, 2019). In summary, clinicians can more accurately select a suitable treatment for patients with ccRCC based on the building blocks of their tumor microenvironment, thus enabling personalized treatment.

We also validated the effect of the genes which constituted the NSS signature on the necroptotic phenotype of ccRCC cells *in vitro*. In cell viability assays, after necroptosis induced by TSZ, excluding BBOX1 and SLC40A1, the other five genes statistically altered the cellular viability. The knockdown of CDH2 resulted in reduced cell viability, whereas PDK4, SLC16A12, TEK, and PLS1 had the opposite effect. Next, we detected the MLKL-dependent necroptosis pathway in cells with different genes downregulated. Through Western blotting, the expression level of key proteins in the MLKL-dependent necroptosis pathway was detected, including RIPK1, RIPK3, MLKL, and p-MLKL. As a result, no changes were found in cells that downregulated these seven genes. This phenomenon may imply that these genes do not regulate the MLKL-dependent pathway. However, the regulation of the MLKL-independent necroptosis pathway was more challenging to validate. This is because the mechanisms underlying the MLKL-independent necroptosis pathway are less well-defined in the literature, making it more difficult to establish its direct links between specific genes. Future studies focusing on these alternative pathways may provide additional insights.

CDH2, known as “neural(N)-cadherin,” was found to participate in the process of epithelial–mesenchymal transition as a cell adhesion molecule (Simon et al., 2024; Nieto et al., 2016; Goossens et al., 2017). In pancreatic ductal adenocarcinoma, Simon et al. (2024) revealed that CDH2 was a specific target of SMAD1; by encoding N-cadherin it can promote migration ability in malignant cells. In addition to its role in necroptosis, CDH2 also plays a role in other processes of programmed cell death (Chen et al., 2023; Gao et al., 2018; Zhou et al., 2023; Ke et al., 2023; Gao et al., 2019; Wang et al., 2025). For example, Chen et al. (2023) found that CDH2 depletion can enhance the susceptibility to ferroptosis by decreasing membrane tension and promoting lipid peroxidation in cells. Gao et al. (2018) showed that downregulation of CDH2 can facilitate apoptosis in prostate cancer. In the context of cuproptosis, Wang et al. (2025) identified the cuproptosis-related gene FDX1 as being significantly associated with CDH2 in colorectal cancer. Additionally, it was demonstrated that CDH2 can promote self-seeding and facilitate the survival of circulating tumor cells in oral cancer. Specifically, soluble CDH2 is able to trigger nature killer (NK) cell functional exhaustion and thus help tumor cells to avoid being killed by NK cells in the circulation (Lou et al., 2022). Moreover, the tumor-infiltrating lymphocyte (TIL) is also an important cell component within the tumor microenvironment. Sun Y. et al. (2021) found that CDH2 can increase the level of PDL1 and IDO1 as well as upregulate the concentration of free fatty acids, enhancing the production of eTreg cells. Taken together, the regulatory role of CDH2 in the tumor immune microenvironment suggests that it could serve as a promising target for anti-tumor immunotherapy.

However, there are still segmental limitations to our study. First, we lack a cohort of ccRCC patients who have received immunotherapy, including ICI therapy, to reveal more about the relationship between NSS and immunotherapy. Second, more *in vivo* and *in vitro* experimental validation is required to elucidate the underlying molecular mechanisms. In summary, we identified hub genes associated with ccRCC necroptosis and used this signature to stratify risk and predict outcomes for ccRCC patients. The universality of the signature is proved by various verifications. We also made nomograms combined with other clinical parameters and analyzed potential therapeutic strategies to prove the clinical applicability of the signature. Therefore, the NSS signature can increase the predictive value for ccRCC patients and make treatment decisions more informed.

## 5 Conclusion

Based on the expression level, mutation, and CNV of necroptotic factors, the subtypes of necroptosis patterns with remarkable prognostic differences were identified in our research. Furthermore, the DEGs between different subtypes were excavated and the key genes associated with prognosis were identified to construct the NSS model. Combined with subsequent survival analysis, immune infiltration analysis, immune checkpoints analysis, drug resistance assessment, and prediction of immune efficacy, NSS is thus certified as a favorable indicator of cancer prognosis and immunotherapy.

## Data Availability

The datasets presented in this study can be found in online repositories. The names of the repository/repositories and accession number(s) can be found in the article/Supplementary Material.
