# Supplementary material for: Development and validation of hierarchical signature for precision individualized therapy based on the landscape associated with necroptosis in clear cell renal cell carcinoma
Source: Front Pharmacol. 2025 Apr 4;16:1470145. doi: 10.3389/fphar.2025.1470145 (PMC12006085; doi:10.3389/fphar.2025.1470145)
Supplement: Supplementary file 1 [file DataSheet1.docx]

Supplementary Material

# Supplementary Figures and Tables

## Supplementary Figures


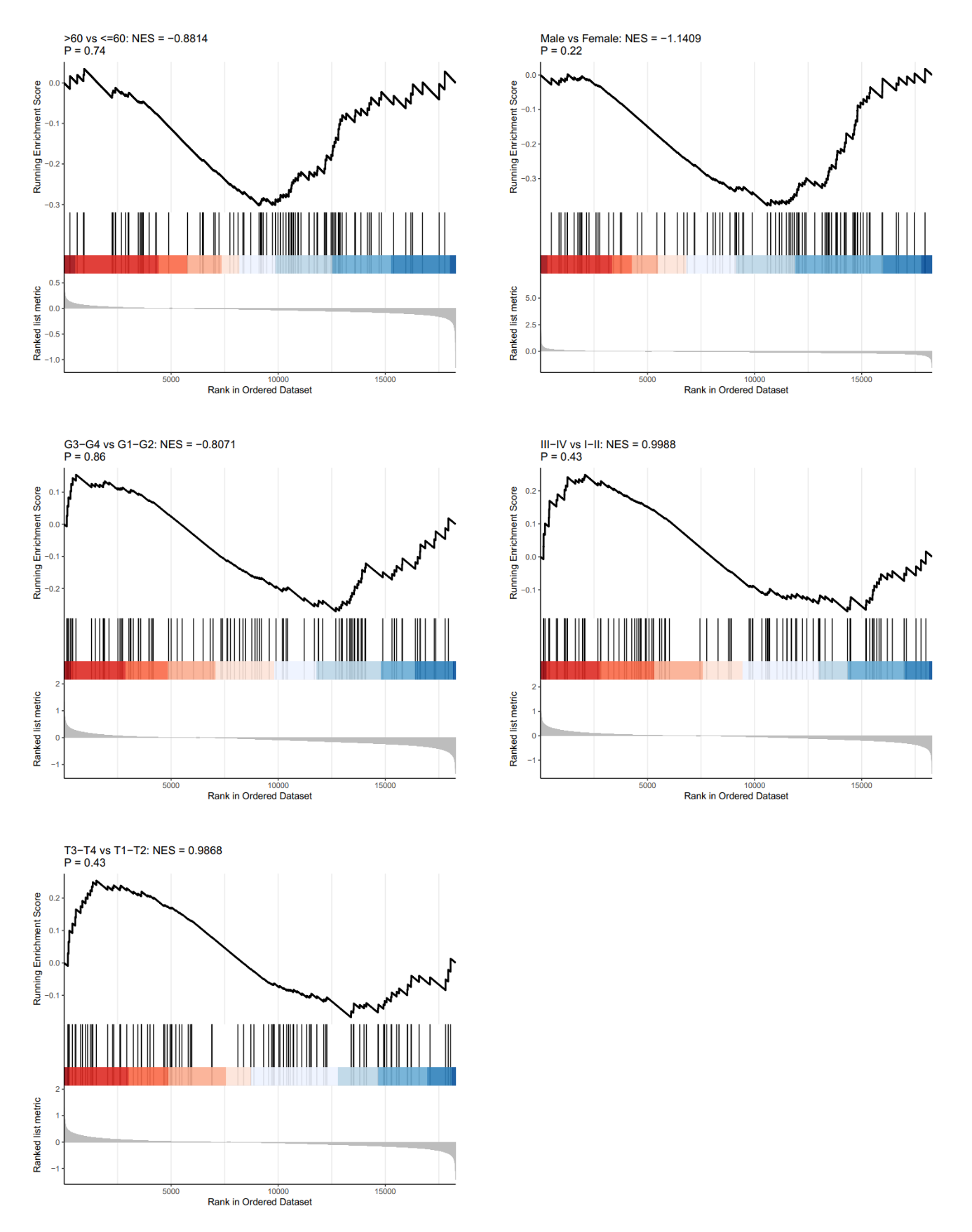


Supplementary Figure 1. GSEA analysis of necroptotic factors in age, sex, Fuhrman grade, TNM stage and T-stage.


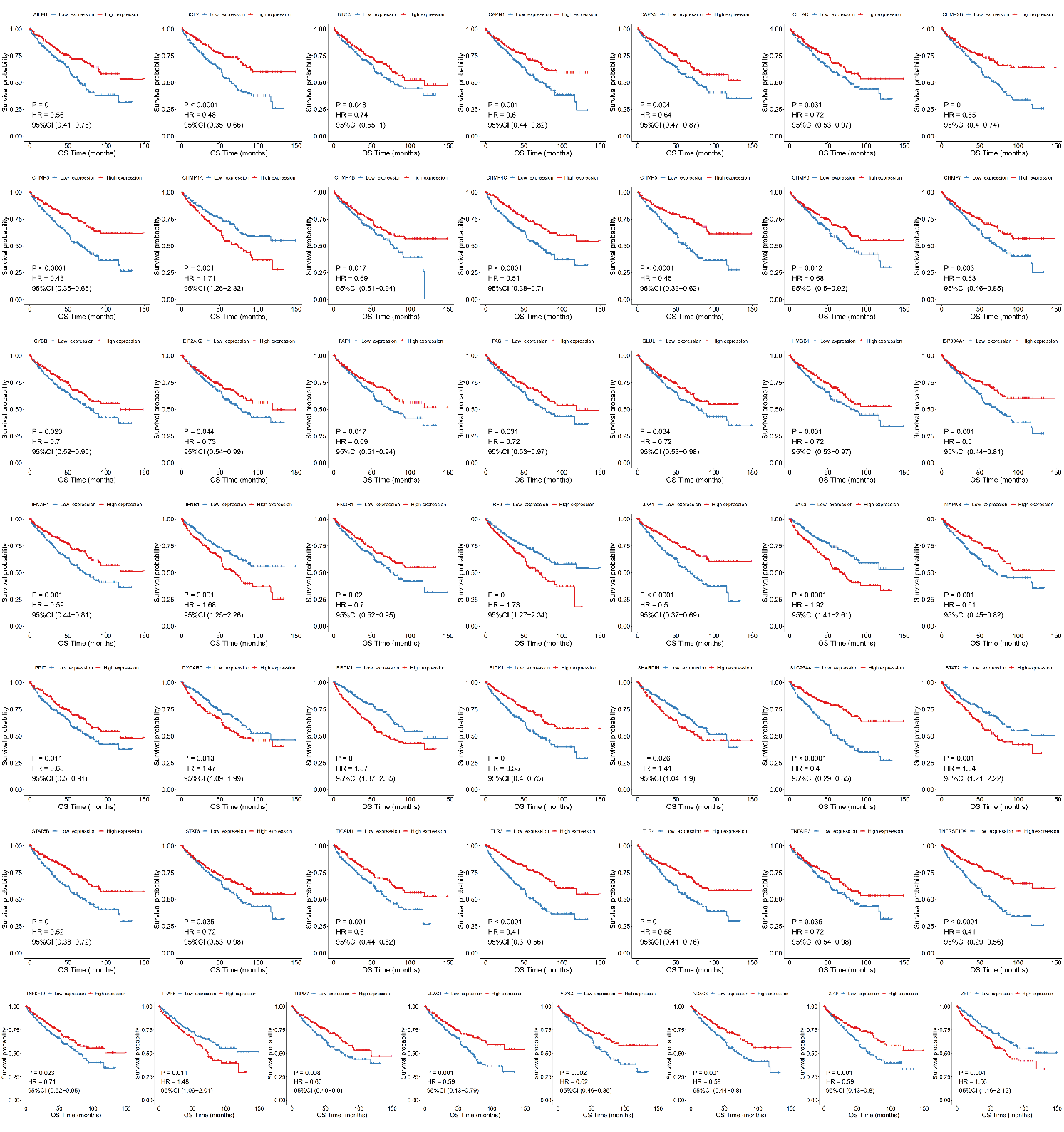


**Supplementary Figure 2.** Kaplan-Meier curves of 50 factors observably correlated with OS.


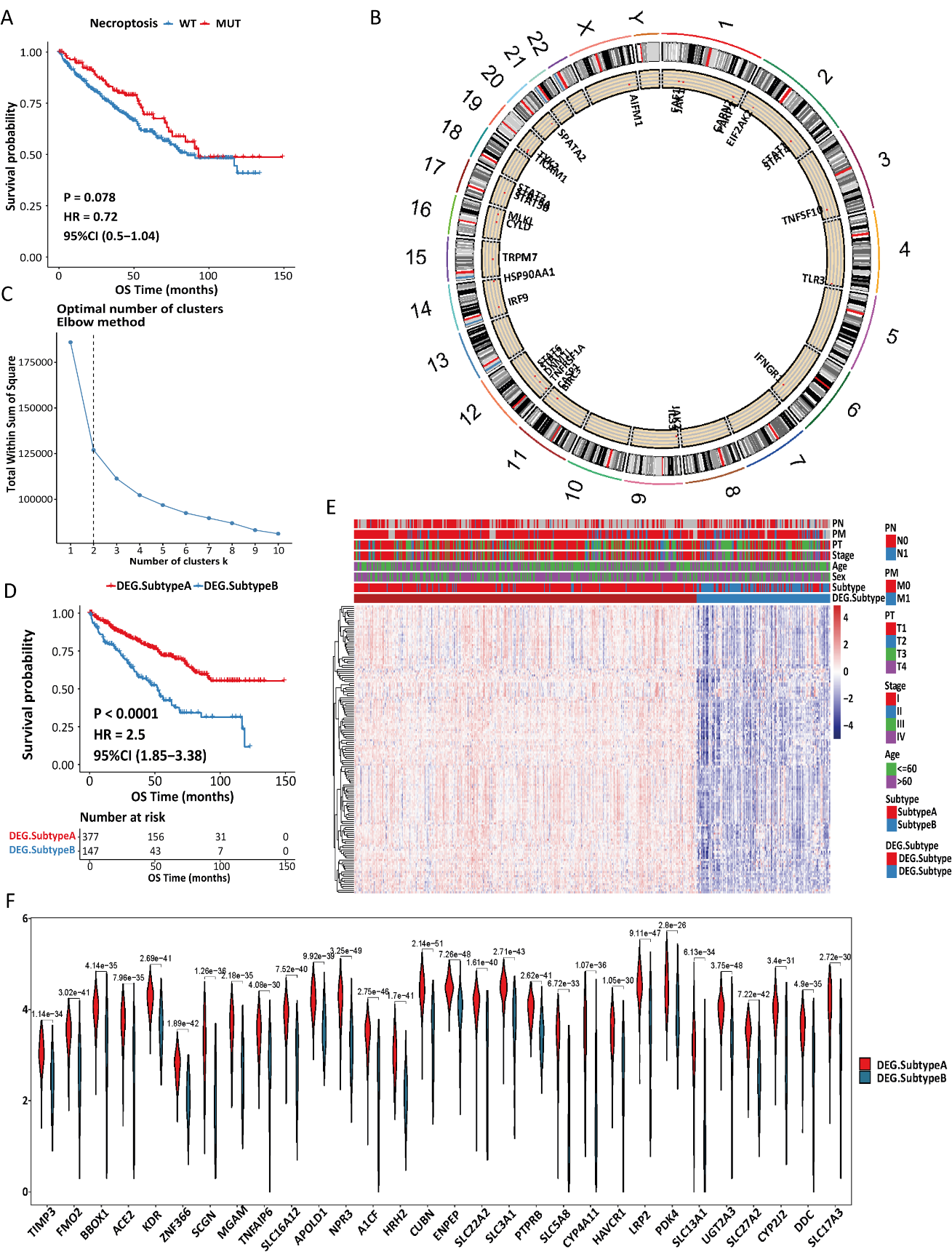


**Supplementary Figure 3. (A)** Kaplan-Meier curves of wildtypes and mutations. **(B)** Location of theses necroptotic factors on the genome. **(C)** K-elbow curve showed 2 clusters. **(D)** Kaplan-Meier curves, **(E,F)** Necroptotic factors expressions between subtypes. (ns, nonsignificant; *, *P* < 0.05; **, *P* < 0.01; ***, *P* < 0.001; ****, *P* < 0.0001.)


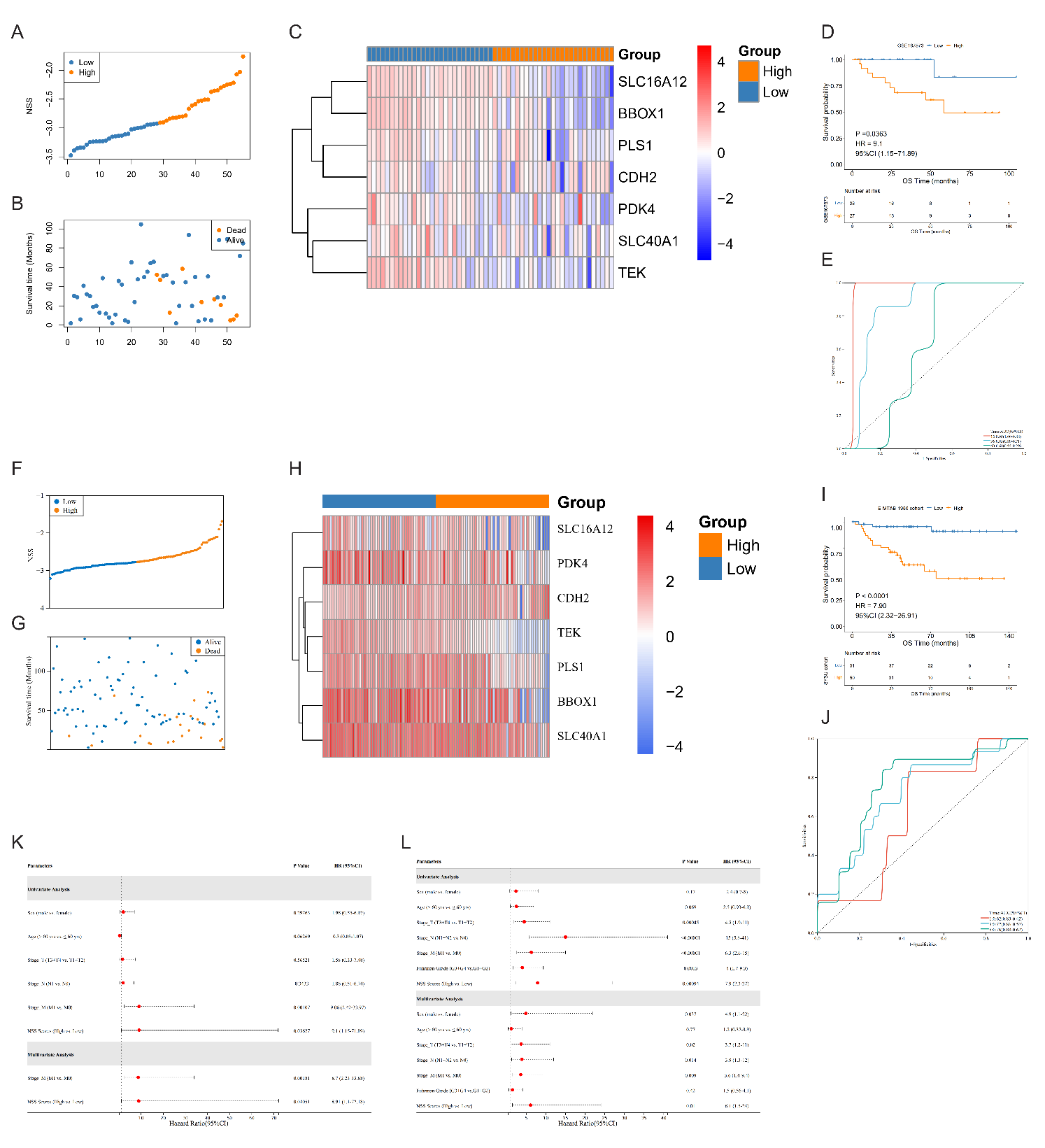


**Supplementary Figure 4.** The validation of NSS. Distribution of NSS scores**(A)** and survival time and status of samples**(B)** in GSE167573. Expressions of model genes**(C)** and Kaplan-Meier curves**(D)** between high- and low-NSS score groups in GSE167573. **(E)** ROC curves of model in GSE167573. F-G, Distribution of NSS scores**(F)** and survival time and status of samples**(G)** in E-MTAB-1980 cohort. Expressions of model genes**(H)** and Kaplan-Meier curves**(I)** between high- and low-NSS score groups in E-MTAB-1980 cohort. **(J)** ROC curves of model in E-MTAB-1980 cohort. **(K,L)** Univariate and multivariate Cox analyses in testing groups.


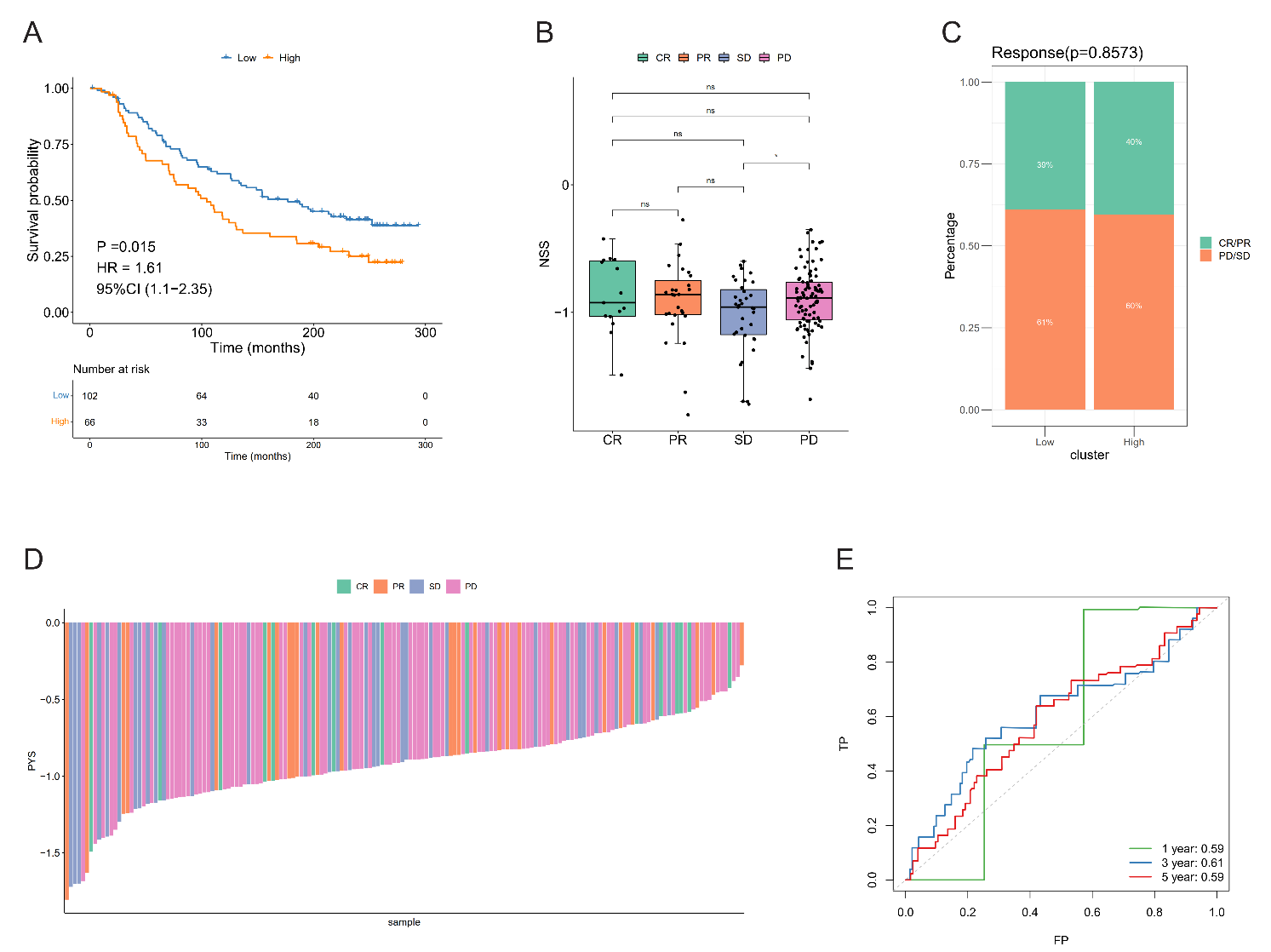


**Supplementary Figure 5.** Differences of overall survival**(A)**, immunotherapy efficacy**(B-D)** between high and low NSS groups. **(E)** ROC curves of NSS in immunotherapy patient cohort.


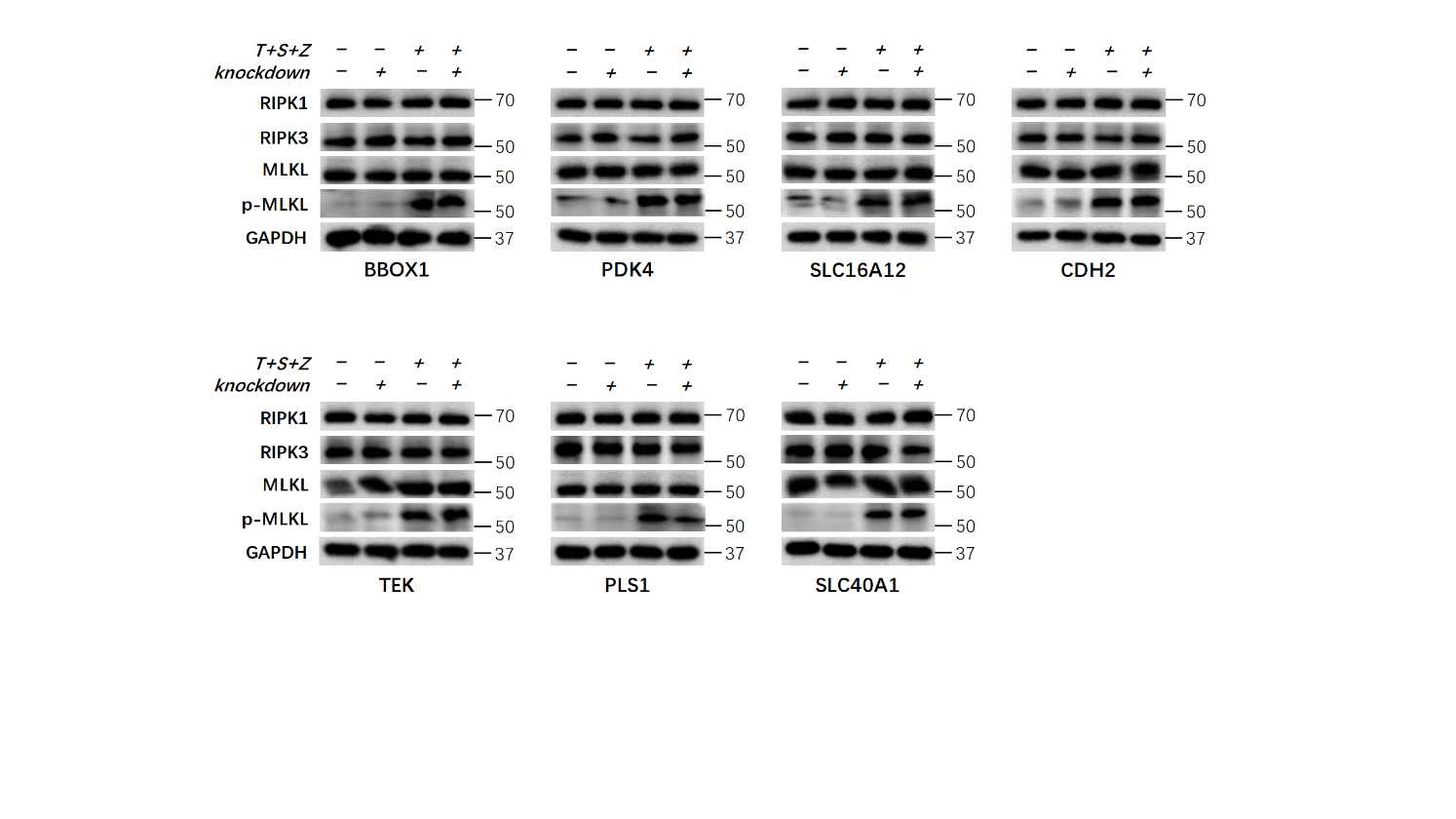


**Supplementary Figure 6.** Necroptosis-related protein expression in cells with and without knockdown of the seven genes.


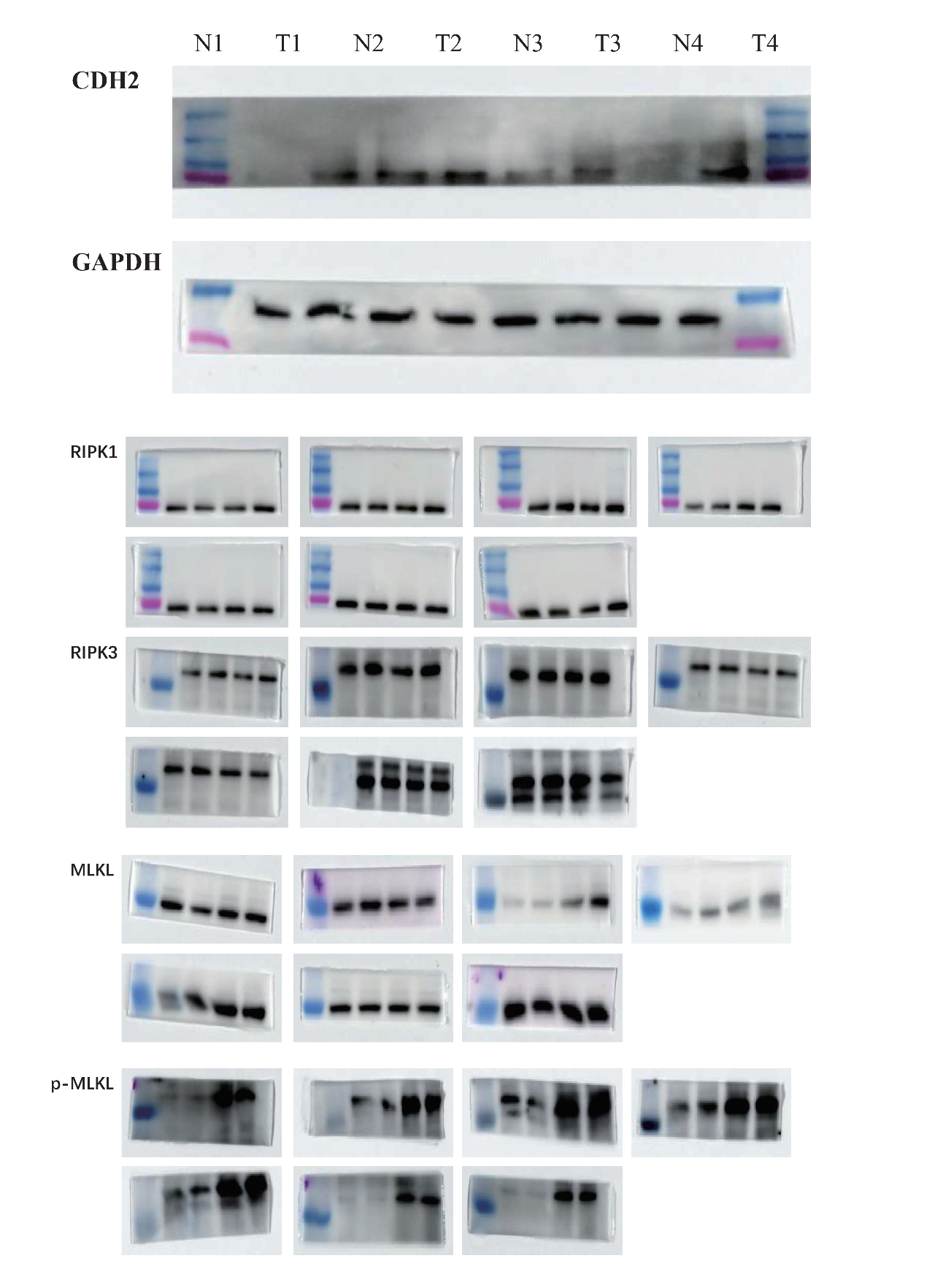


**Supplementary Figure 7.** The original full-length gels involved in article.

## Supplementary Tables

**Supplementary Table 1. 97 necroptosis-related genes in necroptotic pathways**

| Pathway | Genes |
| --- | --- |
| Necroptosis | ALOX15 |
| Necroptosis | FTH1 |
| Necroptosis | CAPN1 |
| Necroptosis | CASP1 |
| Necroptosis | GLUL |
| Necroptosis | BAX |
| Necroptosis | BCL2 |
| Necroptosis | FADD |
| Necroptosis | RIPK1 |
| Necroptosis | TNF |
| Necroptosis | TNFRSF1A |
| Necroptosis | TRADD |
| Necroptosis | TRAF2 |
| Necroptosis | PPIA |
| Necroptosis | CAPN2 |
| Necroptosis | HSP90AA1 |
| Necroptosis | IL1A |
| Necroptosis | FASLG |
| Necroptosis | FAS |
| Necroptosis | CASP8 |
| Necroptosis | MAPK8 |
| Necroptosis | JAK2 |
| Necroptosis | CAMK2B |
| Necroptosis | IL1B |
| Necroptosis | IFNG |
| Necroptosis | STAT3 |
| Necroptosis | IRF9 |
| Necroptosis | TNFSF10 |
| Necroptosis | TNFRSF10A |
| Necroptosis | TNFRSF10B |
| Necroptosis | CFLAR |
| Necroptosis | XIAP |
| Necroptosis | BID |
| Necroptosis | AIFM1 |
| Necroptosis | TRPM7 |
| Necroptosis | IFNAR1 |
| Necroptosis | IFNAR2 |
| Necroptosis | IFNGR1 |
| Necroptosis | IFNGR2 |
| Necroptosis | TLR3 |
| Necroptosis | TICAM2 |
| Necroptosis | IFNA17 |
| Necroptosis | IFNB1 |
| Necroptosis | TICAM1 |
| Necroptosis | VDAC1 |
| Necroptosis | SLC25A4 |
| Necroptosis | PPID |
| Necroptosis | CYLD |
| Necroptosis | RIPK3 |
| Necroptosis | MLKL |
| Necroptosis | TRAF5 |
| Necroptosis | TLR4 |
| Necroptosis | RBCK1 |
| Necroptosis | HMGB1 |
| Necroptosis | JAK1 |
| Necroptosis | JAK3 |
| Necroptosis | TYK2 |
| Necroptosis | STAT1 |
| Necroptosis | STAT2 |
| Necroptosis | STAT4 |
| Necroptosis | STAT5A |
| Necroptosis | STAT5B |
| Necroptosis | STAT6 |
| Necroptosis | TNFAIP3 |
| Necroptosis | RNF31 |
| Necroptosis | CHMP2A |
| Necroptosis | CHMP2B |
| Necroptosis | CHMP3 |
| Necroptosis | CHMP4A |
| Necroptosis | CHMP4B |
| Necroptosis | CHMP6 |
| Necroptosis | VPS4A |
| Necroptosis | CHMP1A |
| Necroptosis | CHMP5 |
| Necroptosis | SMPD1 |
| Necroptosis | PYCARD |
| Necroptosis | NLRP3 |
| Necroptosis | ZBP1 |
| Necroptosis | IL33 |
| Necroptosis | FTL |
| Necroptosis | SQSTM1 |
| Necroptosis | VDAC2 |
| Necroptosis | VDAC3 |
| Necroptosis | CHMP7 |
| Necroptosis | PGAM5 |
| Necroptosis | BIRC2 |
| Necroptosis | BIRC3 |
| Necroptosis | EIF2AK2 |
| Necroptosis | PLA2G4A |
| Necroptosis | DNM1L |
| Necroptosis | SPATA2 |
| Necroptosis | FAF1 |
| Necroptosis | SHARPIN |
| Necroptosis | CYBB |
| Necroptosis | USP21 |
| Necroptosis | PARP1 |
| Necroptosis | CHMP4C |

**Supplementary Table 2. The primers and siRNAs sequences used in the study.**

| **Substances** | **Sequences (5′-3′)** |
| --- | --- |
| **Primers** |  |
| GAPDH Forward | GGAGCGAGATCCCTCCAAAAT |
| GAPDH Reverse | GGCTGTTGTCATACTTCTCATGG |
| BBOX1 Forward | ATGGCTTGTACCATCCAAAAGG |
| BBOX1 Reverse | CGGACAGTTGTCTCTCAACCATA |
| PDK4 Forward | GGAGCATTTCTCGCGCTACA |
| PDK4 Reverse | ACAGGCAATTCTTGTCGCAAA |
| SLC16A12 Forward | TCACTCAGGATTACGCACAAAC |
| SLC16A12 Reverse | TCCCACTTGACAGGATAAATGGT |
| CDH2 Forward | TCAGGCGTCTGTAGAGGCTT |
| CDH2 Reverse | ATGCACATCCTTCGATAAGACTG |
| TEK Forward | TTAGCCAGCTTAGTTCTCTGTGG |
| TEK Reverse | AGCATCAGATACAAGAGGTAGGG |
| PLS1 Forward | ACAAGAGGGAAGGGATTACTGC |
| PLS1 Reverse | AGATGCTTACAGTCAGGGTCATT |
| SLC40A1 Forward | CTACTTGGGGAGATCGGATGT |
| SLC40A1 Reverse | CTGGGCCACTTTAAGTCTAGC |
| **siRNAs** |  |
| sh-NC | GCGACGATCTGCCTAAGAT |
| si-BBOX1 | CGATGAGCATTACAGTGAA |
| si-PDK4 | GACCGCCTCTTTAGTTATA |
| si-SLC16A12 | CACTCAGGATTACGCACAA |
| si-CDH2 | GTAGCTAATCTAACTGTGA |
| si-TEK | GGAATGACATCAAATTTCA |
| si-PLS1 | GTTGTACAGTGGTCAACAT |
| si-SLC40A1 | GGATGGGTCTCCTACTACA |

**Supplementary Table 3. Univariate and multivariate Cox regression analyses on OS in testing groups.**

| **E-MTAB-1980 cohort** | | | | |
| --- | --- | --- | --- | --- |
| **Parameters** | **Univariate Analysis** | | | |
|  | **P Value** | **HR** | **Low 95%CI** | **High 95%CI** |
| Sex (male vs. female) | 0.17 | 2.4 | 0.7 | 8 |
| Age (> 60 yrs vs. ≤ 60 yrs) | 0.069 | 2.5 | 0.93 | 6.9 |
| Stage_T (T3+T4 vs. T1+T2) | 0.00045 | 4.5 | 1.9 | 11 |
| Stage_N (N1+N2 vs. N0) | <0.00001 | 15 | 5.8 | 41 |
| Stage_M (M1 vs. M0) | <0.00001 | 6.3 | 2.6 | 15 |
| Fuhrman Grade (G3+G4 vs.G1+G2) | 0.0013 | 4 | 1.7 | 9.3 |
| NSS Scores (High vs. Low) | 0.00094 | 7.9 | 2.3 | 27 |
| **Parameters** | **Multivariate Analysis** | | | |
|  | **P Value** | **HR** | **Low 95%CI** | **High 95%CI** |
| Sex (male vs. female) | 0.037 | 4.9 | 1.1 | 22 |
| Age (> 60 yrs vs. ≤ 60 yrs) | 0.77 | 1.2 | 0.37 | 3.9 |
| Stage_T (T3+T4 vs. T1+T2) | 0.02 | 3.7 | 1.2 | 11 |
| Stage_N (N1+N2 vs. N0) | 0.014 | 3.9 | 1.3 | 12 |
| Stage_M (M1 vs. M0) | 0.009 | 3.6 | 1.4 | 9.4 |
| Fuhrman Grade (G3+G4 vs.G1+G2) | 0.42 | 1.5 | 0.56 | 4.1 |
| NSS Scores (High vs. Low) | 0.01 | 6.1 | 1.5 | 24 |
| **GSE167573** | | | | |
| **Parameters** | **Univariate Analysis** | | | |
|  | **P Value** | **HR** | **Low 95%CI** | **High 95%CI** |
| Sex (male vs. female) | 0.29763 | 1.96 | 0.55 | 6.97 |
| Age (> 60 yrs vs. ≤ 60 yrs) | 0.06269 | 0.3 | 0.08 | 1.07 |
| Stage_T (T3+T4 vs. T1+T2) | 0.56521 | 1.58 | 0.33 | 7.48 |
| Stage_N (N1 vs. N0) | 0.3433 | 1.86 | 0.51 | 6.74 |
| Stage_M (M1 vs. M0) | 0.00107 | 9.06 | 2.42 | 33.97 |
| NSS Scores (High vs. Low) | 0.03627 | 9.1 | 1.15 | 71.89 |
| **Parameters** | **Multivariate Analysis** | | | |
|  | **P Value** | **HR** | **Low 95%CI** | **High 95%CI** |
| Stage_M (M1 vs. M0) | 0.00181 | 8.7 | 2.23 | 33.88 |
| NSS Scores (High vs. Low) | 0.04051 | 8.91 | 1.1 | 72.18 |

Abbreviations: OS, overall survival; HR, hazard ratio; CI, confidence interval.

**Supplementary Table 4. List of abbreviations and corresponding full names**

| **Abbreviation** | **Full name** |
| --- | --- |
| ARHGAP42 | rho GTPase activating protein 42 |
| ATR | ataxia telangiectasia and Rad3-related protein |
| AUC | area under curve |
| BBOX1 | γ-butyrobetaine hydroxylase 1 |
| BCL2 | B cell lymphoma 2 |
| BID | BH3 interacting domain death agonist |
| CB | clinical benefit |
| CCK8 | cell counting kit-8 |
| ccRCC | clear cell renal cell carcinoma |
| CD86 | cluster of differentiation 86 |
| CDH2 | cadherin 2 |
| CDKN2A | cyclin dependent kinase inhibitor 2A |
| CDKN2B | cyclin dependent kinase inhibitor 2B |
| CHMP3 | charged multivesicular body protein 3 |
| CHMP4C | charged multivesicular body protein 4C |
| CIBERSORT | Cell-type Identification By Estimating Relative Subsets Of RNA Transcripts |
| CNV | copy number variation |
| CSMD3 | CUB and Sushi multiple domains 3 |
| DAMPs | damage-associated molecular patterns |
| DEGs | differentially expressed genes |
| DTWD2 | DTW domain containing 2 |
| ERK1 | extracellular signal-regulated kinase 1 |
| ERK2 | extracellular signal-regulated kinase 2 |
| ESTIMATE | Estimation of STroma and Immune cells in MAlignant Tumor tissues using Expression data |
| eTreg | effector regulatory T cell |
| FC | fold change |
| FDR | false discovery rate |
| FPK | fragments per kilobase million |
| GAPDH | glyceraldehyde-3-phosphate dehydrogenase |
| GDSC | Genomics of Drug Sensitivity in Cancer |
| GEO | Gene Expression Ominibus |
| GISTIC2 | Genomic Identification of Significant Targets in Cancer 2 |
| GSEA | Gene Set Enrichment Analysis |
| GSVA | Gene Set Variation Analysis |
| HEG1 | heart development protein with EGF like domains 1 |
| IC50 | half maximal inhibitory concentration |
| ICIs | immune checkpoint inhibitor |
| IDO1 | indoleamine 2,3-dioxygenase 1 |
| IFNRs | interferon receptors |
| JAK1 | Janus kinase 1 |
| JNK | c-Jun N-terminal kinase |
| KEGG | Kyoto Encyclopaedia of Genes and Genomes |
| KLF10 | KLF transcription factor 10 |
| KM | Kaplan-Meier |
| LASSO | Least Absolute Shrinkage and Selection Operator |
| MAPK8 | mitogen-activated protein kinase 8 |
| MFAP3 | microfibril associated protein 3 |
| MLKL | mixed lineage kinase domain-like protein |
| MUT | mutation |
| NCB | no clinical benefit |
| NK | nature killer |
| NRGs | necroptosis-related genes |
| NSS | necroptosis scoring system |
| OS | overall survival |
| PBRM1 | polybromo 1 |
| PDK4 | pyruvate dehydrogenase kinase 4 |
| PDL1 | programmed death-ligand 1 |
| PDL2 | programmed death-ligand 2 |
| PFS | progression-free survival |
| PLS1 | plastin 1 |
| PTPRD | protein tyrosine phosphatase receptor type D |
| qRT-PCR | quantitative reverse transcription polymerase chain reaction |
| RCCs | renal cell carcinomas |
| RIPK1 | receptor-interacting protein kinase 1 |
| RIPK3 | receptor-interacting protein kinase 3 |
| RNAi | RNA interference |
| ROC | Receiver Operating Characteristic |
| RSK | ribosomal S6 kinase |
| SGPP2 | sphingosine-1-phosphate phosphatase 2 |
| siRNA | small interfering RNAs |
| SLC16A12 | solute carrier family 16 member 12 |
| SLC40A1 | solute carrier family 40 member 1 |
| SLCO2A1 | solute carrier organic anion transporter family member 2A1 |
| ssGSEA | single-sample Gene Set Enrichment Analysis |
| STAT2 | signal transducer and activator of transcription 2 |
| STRING | Search Tool for the Recurring Instances of Neighbouring Genes |
| TCGA | The Cancer Genome Atlas |
| TEK | TEK receptor tyrosine kinase |
| TIL | tumor-infiltrating lymphocyte |
| TLRs | toll like receptors |
| TLR3 | toll like receptor 3 |
| TNF | tumor necrosis factor |
| TNFRs | tumor necrosis factor receptors |
| TPM | transcripts per million |
| TSZ | TNF-α, Smac mimetic, and z-VAD |
| VHL | von Hippel-Lindau tumor suppressor |
| WT | wild-type |
